# ELMO1 signaling is a promoter of osteoclast function and bone loss

**DOI:** 10.1038/s41467-021-25239-6

**Published:** 2021-08-17

**Authors:** Sanja Arandjelovic, Justin S. A. Perry, Ming Zhou, Adam Ceroi, Igor Smirnov, Scott F. Walk, Laura S. Shankman, Isabelle Cambré, Suna Onengut-Gumuscu, Dirk Elewaut, Thomas P. Conrads, Kodi S. Ravichandran

**Affiliations:** 1 Center for Cell Clearance, Department of Microbiology, Immunology, and Cancer Biology and Carter Immunology Center, University of Virginia, Charlottesville, VA USA; 2 Inova Schar Cancer Institute, Inova Center for Personalized Health, Fairfax, VA USA; 3 Inflammation Research Centre, VIB, and the Department of Biomedical Molecular Biology, Ghent Univeristy, Ghent, Belgium; 4 Brain Inflammation and Glia Center and Department of Neuroscience, University of Virginia, Charlottesville, VA USA; 5 Center for Public Health Genomics, University of Virginia, Charlottesville, VA USA

**Keywords:** RNA sequencing, Cell signalling, Mechanisms of disease, Bone, Bone quality and biomechanics

## Abstract

Osteoporosis affects millions worldwide and is often caused by osteoclast induced bone loss. Here, we identify the cytoplasmic protein ELMO1 as an important ‘signaling node’ in osteoclasts. We note that *ELMO1* SNPs associate with bone abnormalities in humans, and that ELMO1 deletion in mice reduces bone loss in four in vivo models: osteoprotegerin deficiency, ovariectomy, and two types of inflammatory arthritis. Our transcriptomic analyses coupled with CRISPR/Cas9 genetic deletion identify *Elmo1* associated regulators of osteoclast function, including cathepsin G and myeloperoxidase. Further, we define the ‘ELMO1 interactome’ in osteoclasts via proteomics and reveal proteins required for bone degradation. ELMO1 also contributes to osteoclast sealing zone on bone-like surfaces and distribution of osteoclast-specific proteases. Finally, a 3D structure-based ELMO1 inhibitory peptide reduces bone resorption in wild type osteoclasts. Collectively, we identify ELMO1 as a signaling hub that regulates osteoclast function and bone loss, with relevance to osteoporosis and arthritis.

## Abstract (web summary)

Osteoporosis and bone fractures affect millions of patients worldwide and are often due to increased bone resorption. Here the authors identify the cytoplasmic protein ELMO1 as an important ‘signaling node’ promoting the bone resorption function of osteoclasts.

## Introduction

Bone tissue turnover occurs throughout life and is vital for the maintenance of nutrient balance and damage repair. Bone loss (osteoporosis) impacts over 200 million people worldwide, with the risk level increasing with age—1 in 3 women and 1 in 5 men over the age of 50 experience bone fractures due to osteoporosis^1^. In addition, bone loss is a hallmark of rheumatoid arthritis, a chronic inflammatory disease of the synovial joints that affects ~1% of the human population^2^. While previous studies have identified multiple molecular players involved in bone loss^3^, including genes and transcription factors that regulate the generation of osteoclasts (osteoclastogenesis)^4^, the current treatment modalities are far from adequate. Further, there is a growing appreciation that targeting the functional activity of osteoclasts may be beneficial^3^, necessitating a better understanding of additional factors that control and contribute to osteoclast-mediated bone loss.

The cytoplasmic adapter protein ELMO1 was originally identified as a component of the machinery that facilitates phagocytosis of apoptotic cells, from nematodes to humans^5,6^. In this context, ELMO1 was shown to associate with DOCK and RAC proteins to mediate cytoskeletal reorganization leading to corpse uptake^5,6^. Further, ELMO1 was also shown to regulate gradient-dependent migration of cells to specific cues^7^. Consistent with its role in cell migration, ELMO1-dependent movement of neutrophils to inflamed joints contributes to arthritis progression^8^. Here, we show that ELMO1 acts as a signaling hub affecting the function of osteoclasts, cells that mediate bone degradation, to promote bone loss in arthritis and osteoporosis.

## Results

### ELMO1 promotes bone loss in models of osteoporosis

**Fig. 1:**
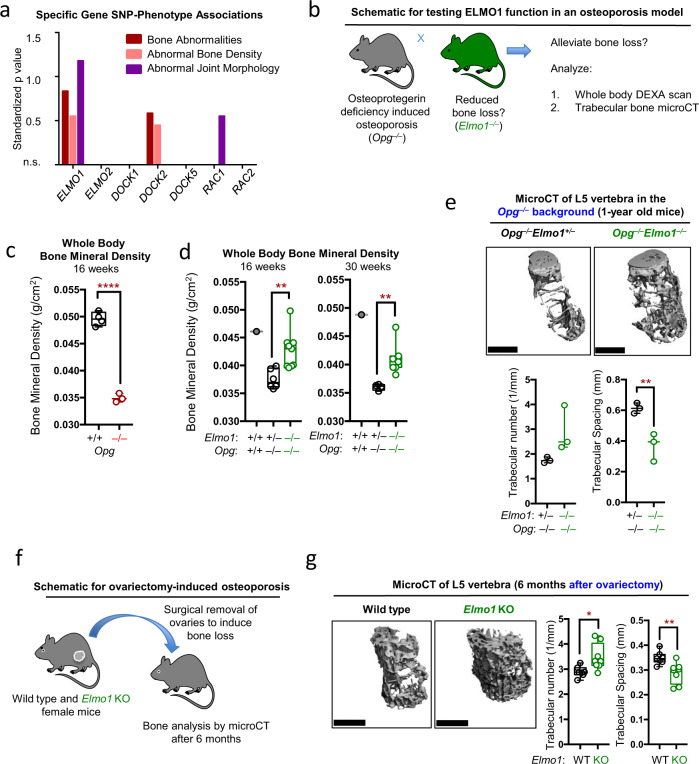
*Elmo1*^*−/−*^ mice have reduced bone erosion in two models of osteoporosis. **a** Single-nucleotide polymorphisms (SNP)-disease and phenotype associations discovered via search of the GWASdb SNP-Disease and six other SNP-phenotype association databases. The data are plotted using a standardized *p* value. Databases used and specific SNPs are indicated in Supplementary Table 1. **b** Schematic for testing ELMO1 function in alleviating bone loss caused by osteoprotegerin (OPG) deficiency. **c** Whole-body bone mineral density in wild type (black, *n* = 4) and *Opg*^*−/−*^ (red, *n* = 3) mice measured by DEXA scanning. Each symbol represents a mouse. Data are presented in a Box and Whiskers format with the box indicating 25th–75th percentile, all data points shown and median indicated. ****p < 0.0001, Student’s *t* test, two-tailed, unpaired. **d** Whole-body bone mineral density in wild type (gray, *n* = 1), *Elmo1*^*+/−*^*Opg*^*−/−*^ (black, *n* = 6 at 16 weeks, *n* = 4 at 30 weeks) and *Elmo1*^*−/−*^*Opg*^*−/−*^ (green, *n* = 9 at 16 weeks, *n* = 7 at 30 weeks) mice measured by DEXA scanning. Each symbol represents a mouse. Data are presented in a Box and Whiskers format with the box indicating 25th–75th percentile, all data points shown and median indicated. **p < 0.01, Student’s *t* test. **e** Representative microcomputed tomography (MicroCT) images of L5 vertebra trabecular bone (top panels) and quantification of the trabecular numbers and spacing (bottom panels) in *Elmo1*^*+/−*^*Opg*^*−/−*^ (black, *n* = 3) and *Elmo1*^*−/−*^*Opg*^*−/−*^ (green, *n* = 3) mice. Scale bar = 1 mm. Each symbol represents a mouse. Data are presented in a Box and Whiskers format with the box indicating 25th–75th percentile, all data points shown, and median indicated. **p < 0.01, Student’s *t* test, two-tailed, unpaired. **f** Schematic for testing ELMO1 function in ovariectomy-induced osteoporosis. **g** Representative MicroCT images of mouse L5 vertebra trabecular bone (left panels) and quantification of the trabecular numbers and spacing (bottom panels) in *Elmo1*^*+/+*^ (WT, black, *n* = 7) and *Elmo1*^*−/−*^ (KO, green, *n* = 8) mice. Scale bar = 1 mm. Data are presented in a Box and Whiskers format with box indicating 25th–75th percentile, all data points shown, and median indicated. *p < 0.05, **p < 0.01, Student’s *t* test, two-tailed, unpaired. Each symbol represents a mouse. Source data are provided as the Source data file.

Examining publicly available databases, we observed a significant correlation between single-nucleotide polymorphisms (SNPs) in *ELMO1, DOCK2*, and *RAC1* genes with various bone and joint abnormalities in humans (Fig. 1a and Supplementary Table 1). To address the role of ELMO1 in bone pathology, we started by analyzing several in vivo mouse models of osteoporosis/bone erosion. The signaling pathway that drives bone resorption (through osteoclast generation and function) includes the receptor RANK and its ligand RANKL^9,10^. Under homeostatic conditions, RANKL activity is kept ‘in check’ by osteoprotegerin (OPG), a soluble decoy receptor for RANKL, acting as an endogenous inhibitor of RANKL. To test whether ELMO1 influences bone loss, we crossed *Elmo1* null mice to OPG-deficient mice (*Opg*^*−/−*^), a mouse model of osteoporosis^11^(Fig. 1b). While OPG-deficient mice exhibited osteoporosis (Fig. 1c), mice double-deficient for *Elmo1* and *Opg* (*Elmo1*^*−/−*^*Opg*^*−/−*^) showed significantly improved bone density at both 16 and 30 weeks of age (Fig. 1d). To address whether the effects of ELMO1 loss can extend beyond 30 weeks, we aged several cohorts of these mice for microCT analysis. *Opg*^*−/−*^ and *Elmo1*^*+/−*^*Opg*^*−/−*^ mice (that are ELMO1 sufficient) exhibit extensive osteoporosis and significant comorbidities, such as bone breaks and vascular calcification^11^, resulting in few mice reaching advanced age. Nonetheless, microCT-based comparison of the vertebral trabecular bone content and density in those littermate mice that reached 1 year of age showed that the trabecular bone loss that was observed in *Elmo1*^*+/−*^*Opg*^*−/−*^ mice was reduced in *Elmo1*^*−/−*^*Opg*^*−/−*^ mice (Fig. 1e, top panels), evidenced by the increased trabecular number and reduced spacing between the trabeculae (Fig. 1e, bottom panels). These data are suggestive of the potential for life-long effects of ELMO1 loss on bone preservation. As a second model, we tested whether loss of ELMO1 can alleviate bone loss in an established model of post-menopausal osteoporosis, i.e., ovariectomy-induced osteoporosis^12^. We surgically removed the ovaries from 8-week old female *Elmo1*^*+/+*^ and *Elmo1*^*−/−*^ mice and evaluated their bone integrity 6 months postsurgery by microCT analysis (Fig. 1f). Compared to the wild-type littermates, we noted significantly increased trabecular number and reduced spacing between the trabeculae in ovariectomized *Elmo1*^*−/−*^ mice (Fig. 1g). These data suggest that ELMO1 contributes to chronic osteoporosis in noninflammatory settings and that ELMO1 loss decreases bone resorption.

### *Elmo1* deletion decreases bone loss in arthritis

**Fig. 2:**
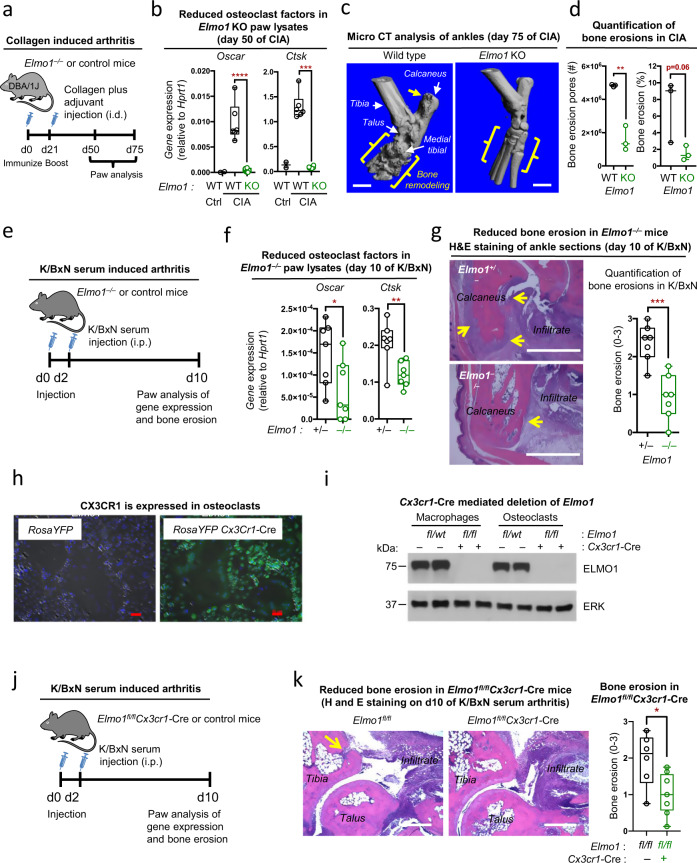
*Elmo1*-deficient mice display reduced bone erosion in two models of arthritis. **a** Schematic of collagen-induced arthritis (CIA) induction in *Elmo*^*−/−*^-DBA/1 J mice. **b** Expression of indicated genes was analyzed in the total paw extracts from either healthy *Elmo1*^*+/+*^-DBA (WT Ctrl, black, *n* = 2), or *Elmo1*^*+/+*^-DBA (WT, black, *n* = 6) and *Elmo*^*−/−*^-DBA (KO, green, *n* = 6) mice on day 50 of CIA by quantitative RT-PCR. Each symbol represents a mouse. Data are presented in a Box and Whiskers format with the box indicating 25th–75th percentile, all data points shown and median indicated. ***p < 0.001, ****p < 0.0001, Student’s *t* test, two-tailed, unpaired. **c** MicroCT images of mouse ankles at the chronic phase of CIA. The significant bone remodeling in *Elmo1*^*+/+*^-DBA mice (wild type), indicated by yellow brackets, is absent in *Elmo1* KO. The yellow arrow denotes bone erosion in the calcaneus. One representative of three mice analyzed is shown. Scale bar = 1 mm. **d** Bone erosions were quantified in the area of the calcaneus of *Elmo1*^*+/+*^-DBA (WT, black, *n* = 3) and *Elmo*^*−/−*^-DBA (KO, green, *n* = 3) mice in the chronic phase of CIA, as described in the Methods. Data are presented in a Box and Whiskers format with the box indicating 25th–75th percentile, all data points shown, and median indicated. **p < 0.01, Student’s *t* test, two-tailed, unpaired. **e** Schematic of the K/BxN serum transfer mediated arthritis induction in *Elmo*^*−/−*^ C57BL/6 mice. **f** Expression of indicated genes was analyzed in the total paw extracts from *Elmo1*^*+/−*^ (black, *n* = 7) and *Elmo1*^*−/−*^ (green, *n* = 7) mice on day 10 after K/BxN serum injection. Each symbol represents a mouse. Data are presented in a Box and Whiskers format with the box indicating 25th–75th percentile, all data points shown, and median indicated. *p < 0.05, **p < 0.01, Student’s *t* test, two-tailed, unpaired. **g** Bone erosions were quantified in the H&E-stained histology sections of mouse ankles from *Elmo1*^*+/−*^ (black, *n* = 7) and *Elmo1*^*−/−*^ (green, *n* = 7) mice on day 10 of K/BxN serum-induced arthritis. Each symbol represents a mouse. Data are presented in a Box and Whiskers format with the box indicating 25th–75th percentile, all data points shown, and median indicated. ***p < 0.001, Student’s *t* test, two-tailed, unpaired. **h** Osteoclasts were differentiated from the bone marrow of *RosaYFP* and *RosaYFP-Cx3cr1*-Cre mice and analyzed (day 6) for YFP protein expression by microscopy. Scale bar, 0.05 mm. A representative of two independent experiments is shown. **i** Confirmation of loss of ELMO1 expression in osteoclasts from *Cx3cr1-Cre-Elmo1*^*fl/fl*^ mice. ELMO1 protein expression in whole cell extracts from macrophages or osteoclasts differentiated from the bone marrow of indicated mice. ERK was used as a protein loading control. A representative of two independent experiments is shown. **j** Schematic of the K/BxN serum transfer mediated arthritis induction in *Elmo*^*fl/fl*^*Cx3cr1-*Cre mice. **k** Bone erosions were quantified in the H&E-stained histology sections of female *Elmo1*^*fl/fl*^*Cx3cr1*-Cre (green, *n* = 8) and *Elmo1*^*fl/fl*^ (black, *n* = 6) on day 10 of K/BxN serum-induced arthritis. Each symbol represents a mouse. Data are presented in a Box and Whiskers format with box indicating 25th–75th percentile, all data points shown, and median indicated. *p < 0.05, Student’s *t* test, two-tailed, unpaired. Source data are provided as the Source data file.

The cartilage and bone erosions are also early hallmarks of the debilitating human inflammatory disease rheumatoid arthritis (RA)^13^. Therefore, we tested whether ELMO1 plays a role in bone loss during arthritis via two mouse models—collagen-induced arthritis and K/BxN serum-induced arthritis (see Methods). To analyze bone remodeling in collagen-induced arthritis (CIA), a model that mimics many features of human rheumatoid arthritis^14^, we back-crossed *Elmo1*^*−/−*^ mice to the DBA/1 J strain (consistently susceptible to collagen-induced arthritis^14^) and immunized them with collagen (Fig. 2a). On day 50 postimmunization (start of the chronic disease stage), paw lysates from arthritic *Elmo1*^*+/+*^DBA mice showed increased expression of osteoclast marker genes *Oscar* and *Ctsk* (Fig. 2b). In contrast, *Oscar* and *Ctsk* expression in *Elmo1*^*−/−*^DBA mice subjected to CIA were not increased (Fig. 2b). We then performed microCT imaging of bone surfaces in the ankles of wild type and *Elmo1*^*−/−*^DBA mice on day 75 of CIA when the chronic phase of the arthritis is usually reached^8^. We noted extensive bone erosion coupled with re-mineralization around the joints of wild-type mice, observed as dents and knobs on the surface of the bone (Fig. 2c and Extended Data Fig. 1a). In contrast, bone surfaces of *Elmo1*^*−/−*^DBA mice were smooth in appearance, suggestive of few erosions and less remodeling events (Fig. 2c and Extended Data Fig. 1a). Bone erosion quantification of the calcaneus confirmed a significant decrease in the number of erosion pores in the *Elmo1*^*−/−*^DBA mice, with the reduction in the percentage of the bone with porous appearance (Fig. 2d and Supplementary Movie 1). Similarly, in the “acute” arthritis model, induced by two injections of K/BxN serum^8^ (Fig. 2e), the paws from *Elmo1*^*−/−*^ mice had significantly decreased expression of osteoclast markers *Oscar* and *Ctsk* (Fig. 2f), and *Elmo1*^*−/−*^ mice exhibited significantly reduced bone erosion (Fig. 2g and Extended Data Fig. 1b).

### ELMO1 function in osteoclasts promotes bone loss in arthritis

In our previous studies, we observed that mice with macrophage lineage-specific deletion of *Elmo1* (*Elmo1*^*fl/fl*^*Cx3cr1*-Cre mice) develop acute K/BxN serum-induced joint inflammation similar to control mice^8^. Osteoclasts are part of the monocytic/macrophage lineage^15^. We first verified that *Cx3cr1*-Cre can target osteoclasts by crossing to the YFP reporter mice to generate *RosaYFP-Cx3cr1*-Cre osteoclasts (Fig. 2h), and that ELMO1 protein expression is lost in *Elmo1*^*fl/fl*^*Cx3cr1*-Cre osteoclasts (Fig. 2i). We then subjected *Elmo1*^*fl/fl*^*Cx3cr1*-Cre mice to K/BxN serum-induced arthritis (Fig. 2j). After verifying that inflammatory scores were not reduced in these mice (Extended Data Fig. 1c), we asked whether there was a difference in bone erosion. Compared to control mice, *Elmo1*^*fl/fl*^*Cx3cr1*-Cre mice showed significantly decreased bone erosions at the peak of acute inflammatory disease (Fig. 2k). Thus, in both the acute and chronic models of arthritis, ELMO1 contributes to the bone erosions, likely due to function in macrophage lineage cells such as osteoclasts.

Bone remodeling involves degradation of the old bone matrix via osteoclasts (of macrophage lineage), followed by new bone mineralization by osteoblasts (mesenchymal origin)^3,15,16^. Osteoclast deficiencies are associated with the development of osteopetrosis, or dense “stone bone,” due to the shift in balance toward bone mineralization^17^. However, healthy *Elmo1*^*+/−*^ and *Elmo1*^*−/−*^ mice do not have significantly increased bone density, when compared to wild-type littermates (Extended Data Fig. 2a). To address the possibility that ELMO1 deficiency results in increased osteoclast numbers, we first quantified osteoclast numbers below the bone growth plate of tibias via staining for TRAP^18^, a marker for mature osteoclasts (Extended Data Fig. 2b). We noted a small but significant increase in TRAP + cells in the bones of *Elmo1*^*−/−*^ mice (Extended Data Fig. 2b). We also tested the serum levels of TRAcP 5b as an additional readout of osteoclast numbers in vivo^19^. Increased TRAcP 5b levels were detected in the serum of *Elmo1*^*−/−*^ mice, compared to controls (Extended Data Fig. 2c, with one *Opg*^*−/−*^ mouse shown as a control for increased osteoclast numbers^11^). On the other hand, the serum levels of the collagen degradation fragments (CTX-I), a measure of osteoclast activity^20,21^, trended toward decreased osteoclast function in ELMO1 deficiency (Extended Data Fig. 2c). We further explored these serum indices of osteoclast number and activity in *Elmo1*^*+/+*^DBA and *Elmo1*^*−/−*^DBA mice under healthy and collagen-induced arthritis conditions. As expected, mice subjected to collagen-induced arthritis displayed increased osteoclast numbers and activity compared to healthy mice (Extended Data Fig. 2d). In these *Elmo1*^*−/−*^DBA mice, we again noted trends toward increased numbers of osteoclasts and decreased activity (Extended Data Fig. 2d). Finally, we tested *Elmo1*^*+/+*^*Opg*^*−/−*^*, Elmo1*^*+/−*^*Opg*^*−/−*^ and *Elmo1*^*−/−*^*Opg*^*−/−*^ mice, in which we noted a significant *Elmo1* dose-dependent decrease in osteoclast activity without a change in osteoclast numbers (Extended Data Fig. 2e). Collectively, these data suggest that loss of ELMO1 could decrease osteoclast activity, which may be compensated by increased osteoclast numbers under baseline conditions (explaining healthy bone appearance unless there are bone loss promoting stimuli). Importantly, osteoblasts established from *Elmo1*^*−/−*^ mice exhibited mineralization comparable to control cultures (Extended Data Fig. 2f). As many diseases with bone demineralization/bone loss associate with augmented osteoclast activity^3,9^, we further tested whether ELMO1 affects osteoclast function.

### ELMO1 promotes bone resorption activity in cultured osteoclasts

**Fig. 3:**
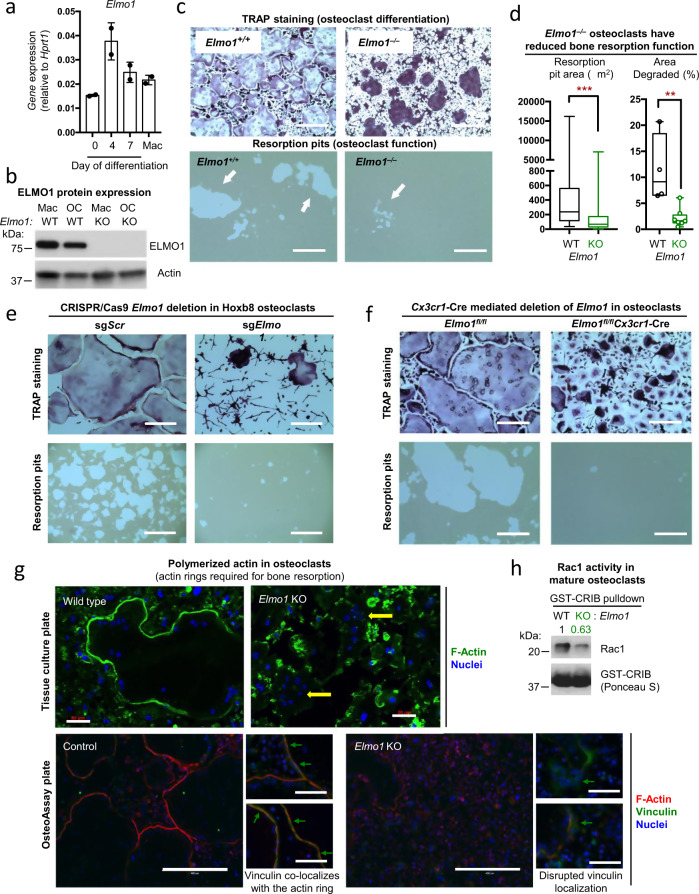
*Elmo1* promotes osteoclast function. **a**, **b** mRNA (scatter plot with bar, mean + /− SD shown) and protein expression for *Elmo1* in osteoclast cultures established from *Elmo1*^*+/–*^ or *Elmo1*^*+/+*^ (WT) and *Elmo1*^*−/−*^ (KO) mice. Actin protein expression is used as a loading control. A representative of three independent experiments is shown. **c**, **d** *Elmo1*^*+/+*^ and *Elmo1*^*−/−*^ osteoclast cultures on day 7 of differentiation were stained for tartrate-resistant acid phosphatase (TRAP, top panels). Scale bar = 0.4 mm. *Elmo1*^*+/+*^ and *Elmo1*^*−/−*^ osteoclasts were tested on OsteoAssay plates to reveal osteoclast resorption pits (bottom panels, arrows). Scale bar = 0.2 mm. One representative of eight experiments is shown. **d** Quantification of the resorption pits in osteoclast cultures from *Elmo1*^*+/+*^ (WT, black) and *Elmo1*^*−/−*^ (KO, green) mice. Data compiled from four WT and eight KO mice are shown. Data are presented in a Box and Whiskers format with a box indicating 25th–75th percentile. Median and Min/Max (left panel) or all points shown (right panel) are indicated. **p < 0.01, ***p < 0.001, Student’s *t* test, two-tailed, unpaired. **e** Hoxb8 sg*Elmo1* and sg*Scr* osteoclast cultures on day 7 of differentiation were stained for TRAP (top panels, scale bar = 0.2 mm) and tested on OsteoAssay plates to reveal osteoclast resorption pits (bottom panels, scale bar = 1 mm). A representative of three experiments is shown. **f** Osteoclasts from *Elmo1*^*fl/fl*^-*Cx3cr1*-Cre mice display a strong defect in bone resorption. Osteoclasts were differentiated from the bone marrow of *Elmo1*^*fl/fl*^ and *Elmo1*^*fl/fl*^-*Cx3cr1*-Cre mice as described in the Methods. On day 5, the cells were fixed and TRAP stained (left panels). Cells grown on OsteoAssay plates were removed on day 5 to reveal the resorption pits (right panels). Scale bars = 0.2 mm. Representative of three experiments is shown. **g** Phalloidin, vinculin, and Hoechst staining (pseudocolored as indicated) of *Elmo1*^*+/+*^ (wild type), *Elmo1*^*+/−*^ (control), and *Elmo1*^*−/−*^ (KO) osteoclasts grown on tissue culture (top panels) or OsteoAssay (bottom panels) plates. Yellow arrows indicate multinuclear cells. Green arrows point to examples of vinculin / F-actin localization. Scale bar = 0.05 mm (top panels), 0.4 mm (bottom large panels), 0.1 mm (bottom small panels). A representative of three experiments is shown. **h** Active Rac1 in whole-cell extracts from WT and *Elmo1*^*−/−*^ (KO) osteoclasts (day 7) were analyzed by immunoblotting (top panels) (Ponceau S staining was used for load control). A representative of two independent experiments is shown. Source data are provided as the Source data file.

Mature osteoclasts are formed by the fusion of osteoclast precursor cells^9^. We established primary cultures of osteoclasts by culturing bone marrow cells with the cytokines M-CSF and RANKL (see Methods). The highest level of *Elmo1* mRNA was seen on day 4 (Fig. 3a) that remained high through day 7 of culture and correlated with ELMO1 protein expression (Fig. 3b). Mature osteoclasts in control cultures were observed as large, multinucleated cells that stained positively for the osteoclast enzyme TRAP (Fig. 3c, top left panel). While *Elmo1*^*−/−*^ cultures also contained TRAP + cells, the morphology of mature *Elmo1*^*−/−*^ osteoclasts looked different, with diminished cell spreading (Fig. 3c, top right panel). Of note, osteoclast precursors from *Elmo1*^*−/−*^ mice could fuse to form multinucleated osteoclasts, and the number of osteoclasts with three or more nuclei was comparable between *Elmo1*^*−/−*^ and control cultures (see Methods and Extended Data Fig. 3a). However, when *Elmo1*^*−/−*^ osteoclasts were plated onto a bone-like matrix that is degraded by osteoclast enzymes, we observed significantly smaller and less frequent “resorption pits” in ELMO1-deficient osteoclast cultures (Fig. 3c, bottom panels and its quantitation in Fig. 3d and Extended Data Fig. 3b), indicating defective osteoclast bone resorption activity.

As another approach, we deleted *Elmo1* in Hoxb8 macrophage precursors through CRISPR/Cas9 and sgRNA (Extended Data Fig. 3c). Osteoclasts differentiated from sg*Elmo1* Hoxb8 cells were TRAP-positive but were again functionally deficient compared to osteoclasts transfected with scrambled sgRNA (sg*Scr*) (Fig. 3e). We also generated Hoxb8 macrophage precursors directly from *Elmo1*^*−/−*^ mice, and osteoclasts derived from these *Elmo1*-deficient precursors also showed a functional defect (Extended Data Fig. 3d). Similarly, osteoclasts from *Elmo1*^*fl/fl*^*Cx3cr1*-Cre mice also showed the deficiency in resorption (Fig. 3f).

### ELMO1 promotes F-actin polymerization in sealing zones

We next tested potential mechanisms by which ELMO1 could be promoting osteoclast function. We confirmed that *Elmo1*^*+/+*^ and *Elmo1*^*−/−*^ cell densities were comparable throughout osteoclast differentiation (Extended Data Fig. 4a). Similarly, we noted no obvious differences in osteoclast differentiation, as evidenced by gene expression of osteoclast-specific markers (Extended Data Fig. 4b). Although osteoclasts belong to the monocytic/macrophage lineage, ELMO1 deficiency did not affect the clearance of apoptotic cells (efferocytosis) by *Elmo1*^*−/−*^ macrophages (as noted previously^22^), suggesting a specificity for ELMO1 in osteoclasts (Extended Data Fig. 5a).

To degrade bone, osteoclasts attach to bone surfaces through the formation of an actin “sealed” ring to sequester and concentrate demineralizing enzymes that in turn, promote bone resorption^23^. When osteoclasts were stained for polymerized actin (F-actin, via phalloidin), control osteoclasts showed strong phalloidin staining along the edges, while the actin rings/sealing zones of *Elmo1*^*−/−*^ osteoclast appeared smaller or incomplete in osteoclasts cultured on tissue culture dishes (Fig. 3g, top panels), bone-like surfaces (Fig. 3g, bottom panels), or bovine bone slices (Extended Data Fig. 5b). While wild-type osteoclasts showed co-localization of F-actin and the cytoskeletal protein vinculin (known to occur at actin sealing zones^23^), such localization was disrupted in *Elmo1* KO osteoclasts (Fig. 3g). We considered the possibility that *Elmo1*^*−/−*^ osteoclasts may lack expression of integrins required for sealing zone maintenance^24^. However, there was in fact even higher expression of integrin β3 in *Elmo1*^*−/−*^ osteoclasts and no change in the expression of integrin β5 (Extended Data Fig. 5c). As ELMO1 is an upstream activator of Rac1 GTPase^5^, and Rac1 affects actin polymerization, we assessed Rac1 activity in wild type and ELMO1-deficient osteoclasts. When we pulled down active Rac1-GTP using an affinity reagent (GST-CRIB), we noted a modest reduction in Rac1 activation in mature *Elmo1*^*−/−*^ osteoclasts (Fig. 3h). Given the strong deficit of ELMO1-deficient osteoclasts in their ability to degrade bone (global *Elmo1* knockout, *Cx3cr1*-Cre driven knockout, or the two types of Hoxb8-derived osteoclasts), and the modest reduction in Rac1 activation, we surmised that ELMO1 likely regulates osteoclast-mediated bone resorption activity in additional ways.

### Elmo1 transcriptional network regulates osteoclast function

**Fig. 4:**
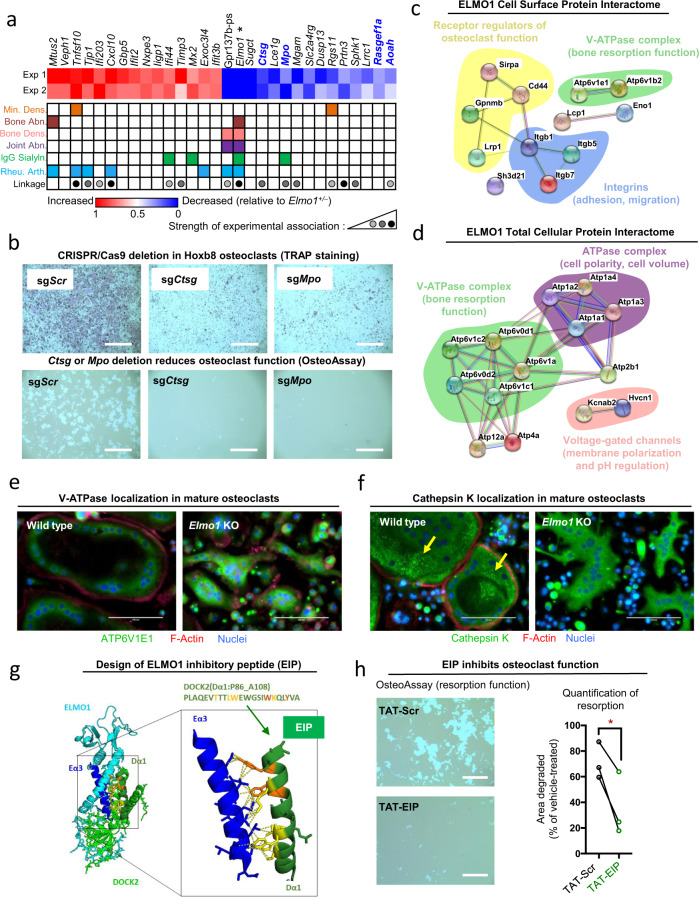
ELMO1 is a signaling hub regulating osteoclast function. **a** Top 15 significantly up and downregulated genes in *Elmo1*^*−/−*^ relative to *Elmo1*^*+/−*^ osteoclasts. Genes are ordered from left to right per magnitude of change (*Elmo1*, used as the positive control, is indicated with an asterisk). SNPs associated with mineral density (Min. Dens.), bone abnormalities (Bone Abn.), bone density (Bone Dens.), joint abnormalities (Joint Abn.), IgG sialylation (IgG Sialyln.), or rheumatoid arthritis (Rheu. Arth.) are denoted by colored squares. Linkage denotes an association of the gene with indicated abnormality. The color of the node denotes the strength of the association (light gray = 1–2, medium gray = 3–5, dark gray = 6–8, corresponding to the number of instances a gene is found as a significant hit in databases listed in Supplementary Table 1). Data from two independent experiments with two mice per group are shown. **b** Hoxb8 cells stably expressing sgRNA targeting cathepsin G (sg*Ctsg*), myeloperoxidase (sg*Mpo*), or scrambled guide RNA (sg*Scr*) were differentiated into osteoclasts for 7 days and stained for TRAP (top panels, scale bar = 0.4 mm). Hoxb8 osteoclast resorption pits were tested on OsteoAssay plates (bottom panels, scale bars = 1 mm). Representative of three independent experiments. **c**, **d** Osteoclast cell surface **c** and total **d** ELMO1 protein interactome. ELMO1 interacting proteins were analyzed via STRING software (see Methods and Supplementary Fig. 3). The clusters of proteins that have been implicated in osteoclast function are highlighted in different colors. **e**, **f** Bone marrow from *Elmo1*^*+/+*^ (wild type) and *Elmo1*^*−/−*^ (KO) mice was differentiated into osteoclasts on OsteoAssay plates for 7 days and the cells were stained as indicated. Arrows indicate cathepsin K puncta. Scale bar = 0.1 mm. Representative of two independent experiments. **g** Design of the inhibitory EIP peptide based on the three-dimensional structure of the ELMO1 (cyan) and DOCK2 (green) binding interface. Interaction of the ELMO1 (Eα3, dark blue) and alpha helices in DOCK2 (Dα1, dark green) is shown. Critical ELMO1 interacting residues of DOCK2 (W102 and Y106, orange), and other contributing residues (yellow) are shown. EIP contains DOCK2 amino acids 86–108. The structure shown (PDB code 3a98) was modeled using PyMOL software. **h** Wild-type osteoclasts were differentiated on OsteoAssay plates with 10 µM EIP (green, *n* = 3) or the scrambled peptide (Scr, black, *n* = 3) from day 3 of differentiation. Scale bar = 0.4 mm. Resorption pits were scored on day 7 and quantified as a % of vehicle-treated cells. Paired symbols represent cultures from individual animals. Data are representative of two independent experiments. *p = 0.0412, Student’s *t* test, two-tailed, paired. Source data are provided as the Source data file.

To assess whether ELMO1 might control other signaling entities in osteoclast function in an unbiased manner, we took complementary transcriptomics and proteomics approaches. First, we examined genes that are affected by *Elmo1* expression in mature osteoclasts via RNAseq. *Elmo1*^*−/−*^ osteoclasts showed altered expression of several genes linked to human diseases (Fig. 4a). These include genes linked to small GTPases and cell motility (such as *Mx2, Rgs11, Mtus2*, and *Rasgef1a*), as well as genes involved in extracellular enzymatic degradation with potential relevance to osteoclast function in bone demineralization (*Mpo, Ctsg, Timp3, Mgam, Prtn3*, and *Aoah*) and inflammation (*Tnfsf10/TRAIL*, *Cxcl10*). Human SNPs in some of these genes are associated with bone mineral density changes, bone, and joint abnormalities, IgG sialylation (linked to bone loss^25^), or rheumatoid arthritis (Fig. 4a, middle rows). This prompted us to analyze the function of select *Elmo1*-regulated genes using the CRISPR/Cas9 gene deletion approach in Hoxb8 osteoclasts. We first targeted cathepsin G (*Ctsg*) and myeloperoxidase (*Mpo*), as these are both enzymes that could potentially play a role in osteoclast functionality. After sgRNA-mediated targeting of *Ctsg* and *Mpo*, we verified the decrease or absence of cathepsin G and myeloperoxidase protein expression (Extended Data Fig. 6a). Hoxb8 osteoclasts differentiated from both *Ctsg* and *Mpo* targeted lines had reduced degradative function, compared to the control sg*Scr* osteoclasts (Fig. 4b, Extended Data Fig. 6b). Of note, even doubling the number of sg*Ctsg* and sg*Mpo* Hoxb8 precursor cells seeded for this assay still resulted in reduced osteoclast activity (Extended Data Fig. 6c). Using the same approach, we also targeted two other *Elmo1*-regulated genes, *Aoah* and *Rasgef1a* (both coding for enzymes), for deletion in Hoxb8 osteoclasts; they both showed reduced osteoclast function (Extended Data Fig. 6d). Importantly, not every enzyme targeted led to decreased osteoclast function, as deletion of *Ero1lb* (decreased in *Elmo1*^*−/−*^ osteoclasts, Supplementary Table 2) enhanced bone resorption activity (Extended Data Fig. 6e). Thus, the transcriptomics approach suggested that ELMO1 is part of a larger signaling/functional network in osteoclasts that regulates bone degradation.

### ELMO1 deficiency disrupts localization of bone degrading enzymes

As a complementary approach, we performed two types of proteomics to define the ELMO1 protein interactome: (i) probing specifically the osteoclast membrane proteins bound to the cytoplasmic ELMO1, and (ii) identifying all of the osteoclast cellular ELMO1 interacting partners. We labeled cell surface proteins in mature osteoclasts by biotinylation and performed ELMO1 immunoprecipitation, enrichment for biotin-labeled proteins (Extended Data Fig. 7a, see Methods for details), and analyzed the “ELMO1 protein interactome” by mass spectrometry and subsequent STRING analysis^26^. ELMO1 associated with cellular receptors that include positive regulators of osteoclast function (such as CD44^27^ and Osteoactivin, encoded by *Gpnmb*^28^), as well as negative regulators (SIRPα^29^ and LRP1^30^) (Fig. 4c and Supplementary Table 3). In addition, ELMO1 engaged two subunits of the vATPase complex (ATP6V1E1/B2) that mediates extracellular acidification and bone demineralization in osteoclasts^31^ (note: although these vATPase subunits are supposed to be cytoplasmic, they likely were enriched through secondary tethering via cytoplasmic ELMO1, or during endocytic processing/recycling of surface receptors). In the second approach, when we analyzed the total cellular ELMO1 protein interactome in osteoclasts, we found an association with additional vATPase complex subunits (such as ATP6V0D1/D2), as well as subunits of complexes that regulate cell polarity and pH regulation, such as ATP1, HVCN1 and KCNAB2^32–34^ (Fig. 4d and Supplementary Table 3). Association of ELMO1 with vATPase subunits was particularly relevant, as the vATPase complex localizes in bone-attached osteoclasts to the specialized membrane area to allow acidification/demineralization near the resorption pit^31^. To investigate this further, we visualized the localization of the vATPase subunit ATP6V1E1 in *Elmo1*^*+/+*^ and *Elmo1*^*−/−*^ osteoclasts plated on the bone-like matrix. In wild-type osteoclasts, ATP6V1E1 localizes close to the osteoclast edges (areas of active resorption) whereas in *Elmo1*^*−/−*^ osteoclasts the membrane-proximal localization for this vATPase subunit was lost with staining redistributed throughout the cell (Fig. 4e). We confirmed the specificity of vATPase staining, as no signal was detected when we used the isotype control antibody (Extended Data Fig. 7b). To test if osteoclast degradative enzymes might also be redistributed in ELMO1-deficient osteoclasts, we examined cathepsin K, a protease that is released into the resorption pits via lysosomal fusion with the plasma membrane^35^. While wild-type osteoclasts show cathepsin K puncta near the outer edges (Fig. 4f, arrows), cathepsin K in *Elmo1*^*−/−*^ osteoclasts was diffused and distributed throughout the cell (Fig. 4f and isotype control in Extended Data Fig. 7c). These data suggest that ELMO1 can influence the localization of other proteins within the signaling network that controls the bone resorption function of osteoclasts.

### ELMO inhibitory peptide disrupts matrix resorption in osteoclasts

In our original identification of the role of ELMO1 in apoptotic cell clearance, we had noted that an extended alpha-helix near the ELMO1 C-terminus is evolutionarily conserved from *C. elegans* to human^6^. We have further determined that this α-helix is required for ELMO1 function in vitro and in vivo^5,7,36,37^. Recently, the 3D-structure of the ELMO1:DOCK2 complex detailed the interaction of the C-terminal alpha-helix in ELMO1 with DOCK2^38^. Based on this, we designed a peptide targeting this alpha-helix to inhibit ELMO1-mediated signaling (denoted ELMO1 inhibitory peptide, or “EIP”) (Fig. 4g). To permit delivery of EIP across the plasma membrane, we fused it to the TAT-sequence (TAT-EIP)^39^. As a control, we used a scrambled EIP peptide sequence fused to TAT (TAT-Scr). When added to osteoclast cultures from wild-type mice, TAT-EIP significantly decreased osteoclast-mediated bone resorption on bone-like matrices, compared to TAT-Scr (Fig. 4h). We also repeated these studies using TAT-Cre as a control and obtained similar results (Extended Data Fig. 7c). Thus, targeting a conserved region within ELMO1 further supports the relevance of an ELMO1-based signaling network in osteoclast function.

## Discussion

Collectively, the data presented here provide insights into osteoclast functional activity and bone remodeling. First, this current work, spanning whole animal models of bone loss to atomic structure-based targeting, identify ELMO1 as an osteoclast-intrinsic positive regulator of bone resorption activity. Second, mechanistic studies identify a larger ELMO1-dependent signaling network, with many of the genes and proteins linked to ELMO1 being independently associated with bone disorders and osteoclast function in humans and in mouse models. One role for ELMO1 and associated proteins is in the proper formation of the actin ring/sealing zone and/or the localization of the bone degrading enzymes for osteoclast activity. Third, paradoxically, osteoclast-derived factors are also needed to attract osteoblasts for new bone formation, and strategies that solely aim to reduce osteoclast numbers have had mixed success in treating osteoporosis; thus, it has been suggested that targeting the resorption “activity” of osteoclasts rather than osteoclast numbers may be more beneficial^3,16^. The ELMO1 signaling network defined here has revealed additional players in osteoclast-mediated matrix degradation, such as cathepsin G and myeloperoxidase. As global ELMO1 null mice are healthy under basal conditions, targeting ELMO1 and/or its signaling nexus components identified here might serve as potential points of therapeutic intervention in both inflammatory and noninflammatory types of bone loss mediated by heightened osteoclast activity.

## Methods

### Mice

C57BL/6 J, DBA/1 J, NOD, *Cx3cr1*-Cre, *Opg*^*−/−*^ and *RosaYFP* mice were obtained from Jackson Laboratories. *Elmo1*^*fl/fl*^ and *Elmo1*^*−/−*^ mice have been described previously^22^. To generate mice with deletion of *Elmo1* in the macrophage lineage, *Elmo1*^*fl/fl*^ mice were crossed to *Cx3cr1*-Cre mice. To generate *Elmo1*^*−/−*^ on the DBA background for collagen-induced arthritis experiments, *Elmo1*^*−/−*^ mice were backcrossed onto the DBA/1 J background for at least five generations. KRN TCR transgenic mice^40^ were a gift from Dr. Diane Mathis at the Harvard Medical School and were bred to NOD mice to obtain the K/BxN mice^41^, which develop progressive spontaneous arthritis. K/BxN serum was collected from 9-weeks old K/BxN mice by cardiac puncture. Serum for TRAcP 5b and CTX-I measurement was collected by cardiac puncture, and ELISA performed following manufacturer’s instructions (Immunodiagnostic Systems, UK). Age- and sex-matched littermate control animals were used for all experiments, and both males and females were assessed. Animals are housed in Allentown ventilated racks with reverse osmosis water through an automatic watering system. They receive ad libitum diet and are kept in a 14:10 light:dark cycle at 72 degrees F and 40% humidity. All animal procedures were approved by and performed according to guidelines of the Institutional Animal Care and Use Committee (IACUC) at the University of Virginia under the protocol #2992.

### Ovariectomy-induced osteoporosis

Eight-weeks old females were anesthetized by isofluorane inhalation, fur on the dorsum was clipped and skin disinfected with surgical iodine and 70% ethanol using sterile swabs. After a 4–7 mm skin/abdominal incisions parallel and ventral to the spine, forceps were used to remove the ovary, while preventing bleeding. The uterine horn and the fat pad were returned to the abdominal cavity. The incision in the abdominal wall was closed with sutures. All mice received subcutaneous injections of an analgesic (Ketoprofen 2–5 mg/kg, every 24 h over 72 h postoperation) and an antibiotic (Baytril 5 mg/kg, every 24 h for 7 days postoperation). Six months after the surgery, mice were euthanized, and vertebral columns were extracted and fixed in 10% formalin (Fisher) for microCT analysis.

### K/BxN serum transfer induced arthritis and collagen-induced arthritis (CIA)

For inducing arthritis using the K/BxN serum, mice were injected with 150 μl of serum from K/BxN mice on days 0 and 2. For collagen-induced arthritis, *Elmo1*^*−/−*^ mice in the DBA background were immunized at the base of the tail by intradermal injection of a 1:1 solution of 10 mM acetic acid containing 100 μg of bovine collagen-II and Complete Freund’s Adjuvant (Sigma) containing 100 μg of heat-killed Mycobacterium tuberculosis H37Ra (BD) and boosted by immunization on day 21. Paw swelling and clinical scores were measured as previously reported^8^. All scoring was done by an investigator blinded to the mouse genotypes.

### Histology

For K/BxN serum transfer induced arthritis and CIA experiments, mice were euthanized at the indicated time points and the paws were fixed in 10% formalin (Fisher). Decalcification, sectioning, paraffin embedding, and hematoxylin and eosin (H&E) staining was performed by HistoTox Labs (Boulder, CO). Histology scoring was performed by an investigator blinded to the mouse genotypes. For inflammation scoring, the following criteria were used—0, none; 1, mild; 2, moderate; 3, severe. For bone erosion scoring, the following criteria were used: 0, no bone erosions observed; 1, mild cortical bone erosion; 2, severe cortical bone erosion without the loss of bone integrity; 3, severe cortical bone erosion with the loss of cortical bone integrity and trabecular bone erosion. For TRAP staining, paws were fixed in phosphate-buffered saline (PBS) containing 4% paraformaldehyde for 72 h at 4 °C, followed by decalcification with 10% EDTA, pH 7.0 for 3 weeks with two weekly changes of buffer. Sectioning and paraffin embedding were performed as above. Deparafinized sections were incubated in 0.2 M Tris, pH9.0 for 1 h at 37 °C, followed by staining using the acid phosphatase, Leukocyte (TRAP) staining kit (Sigma), following manufacturer’s instructions. For RNA and protein isolation, the paws were snap frozen in liquid nitrogen at indicated time points and subjected to mechanical disruption using a tissue pulverizer (Spectrum Laboratories, Inc.).

### Dual energy X-ray absorptiometry (DEXA) scan analysis

Mice were anesthetized using a sterile saline solution containing 20 mg/ml ketamine and 2 mg/ml xylazine, and the whole body was scanned on a Piximus 2 (GE Medical Systems Lunar Corp.) DEXA scanner and analyzed using Lunar Piximus 2.10 software.

### Microcomputed tomography (MicroCT) analysis

MicroCT analyses were performed on isolated tissues using a SCANCO VivaCT 40 scanner (Scanco). Mouse legs or vertebral columns were scanned in 10–15 ml tubes filled with PBS containing 4% paraformaldehyde at 55 kV and 145 μA, with a voxel size of 12.5 μm. Projection images were reconstructed, and three-dimensional visualization was performed using Scanco IPL V5.15. For the trabecular bone analysis, the regions of interest were defined immediately below and between the bone growth plates. The whole hind paw ankles were scanned and analyzed. For bone erosion quantification, samples were scanned on HECTOR (developed in collaboration with XRE, www.xre.be)^42^, using a directional X-ray source set at 130 kV and 10 Watt beam power with a 1 mm Aluminium filtration. The detector was a Perkin–Elmer flat-panel measuring 40 × 40 cm, with a pixel pitch of 200 μm. A total of 2000 projections of 1-s exposure time each were recorded. The resulting scan images have a voxel size of 4.5 μm. The obtained projection images of all scans were reconstructed using Octopus Reconstruction^43^, software originally developed by UGCT based on the standard FDK algorithm for reconstruction of CT data. 3D visualizations were made using the commercial rendering software VGStudioMAX (Volume Graphics). Analysis of calcaneus surface erosion was performed using a custom script in ImageJ. Within a 50 μm thick outer layer of the calcaneus, surface erosions were digitally filled up to achieve a smooth surface. The volume of this filling, representing the surface erosion volume, was then measured and expressed as a ratio to the total bone volume. Our script utilized the “Optimise Threshold” plugin from BoneJ for converting the greyscale images to a binary format. Vertebral columns from ovariectomized mice were scanned at Scanco Medical, using a Scanco Medical mCT 40. All scans were conducted using an x-ray energy level of 55 kvp and a voxel resolution of 8 μm. 3d-morphometric analysis was conducted using the built-in Scanco software on a volume of interest, chosen to include the trabecular bone region of the vertebral body, with a threshold of 290.

### Macrophage and osteoclast cultures

For bone marrow-derived macrophage and osteoclast cultures, femurs were removed from 6–8 weeks old mice, flushed with sterile serum-free α-MEM (Corning), and single-cell suspensions were prepared using the 70 μm cell strainer (Fisher). Cells were spun down and cultured on petri dishes in α-MEM containing 10% FBS and 1% penicillin/streptomycin/glutamine at 37 °C and 5% carbon dioxide for 16 h. Osteoclasts were differentiated as previously described^44^. Briefly, suspension cells were collected and cultured on petri dishes as above with added 10% L929 cell (ATCC, # CCL-1) conditioned media as a source of macrophage-colony-stimulating factor. After 3 days, cells attached to the dishes were collected and 50,000 cells were plated on tissue culture coated 24-well plates (Corning), OsteoAssay plates (Corning), bovine bone slices (Immunodiagnostic Systems), or glass coverslips (Fisher), and cultured for an additional 7–14 days with 50 ng/ml of GST-RANKL (a gift from Dr. Steven Teitelbaum, Washinton University in St. Louis, MO) to obtain osteoclasts, or vehicle control. The ELMO1 inhibitory peptide (EIP), comprised of the TAT peptide (GRKKRRQRRRPQ) fused to the N-terminus of the ELMO1-binding DOCK2 amino acids 86–108^38^, the scrambled peptide (TAT peptide fused to the scrambled DOCK2 peptide sequence), and the TAT-Cre (TAT peptide fused to the Cre recombinase enzyme^39^) were synthesized at Genscript. All peptides were added on day 3 of osteoclast differentiation at 10μM concentration and replenished daily until day 7. TRAP staining was performed using acid phosphatase, leukocyte (TRAP) staining kit (Sigma), following manufacturer’s instructions. To reveal osteoclast resorption pits, cells plated on OsteoAssay plates were bleached, and the plates dried following manufacturer’s instructions. Resorption pits were quantified using Image J. Cell Profiler v3.1.9 was used to create a pipeline to automatically analyze eroded regions. Color images were converted to grayscale and thresholded using a global three-class Otsu algorithm before pits were identified as primary objects. Primary objects were measured, and the sum of areas was divided by the total number of pixels to determine the percent area eroded.

### Osteoblast culture

Femurs were removed from 6–8 weeks old mice, flushed with sterile serum-free α-MEM (Corning) and single-cell suspensions were prepared using the 70 μm cell strainer (Fisher). Cells were spun down, and 1–2 million cells were cultured per well in 6-well tissue culture coated dishes in α-MEM containing 10% FBS and 1% penicillin/streptomycin/glutamine at 37 °C and 5% carbon dioxide. Suspension cells were removed after 16 h, and adherent cells were grown to confluence. Osteoblast differentiation was induced by culturing in α-MEM containing 10% FBS, 1% penicillin/streptomycin/glutamine, 50 μm ascorbic acid (Sigma), 10 mM β-glycerophosphate (Sigma), and 10 nM dexamethasone (Calbiotech)^45^. After 14–21 days of differentiation, cells were fixed with 10% neutral buffered formalin (Fisher), stained with 40 mM Alizarin Red (Sigma) pH 5.6, and imaged on an EVOS FL Auto (Thermo Fisher) and analyzed using the accompanying software.

### Peritoneal macrophage efferocytosis of apoptotic thymocytes

Eight-weeks old *Elmo1*^*+/+*^ (*n* = 3) and *Elmo1*^*−/−*^ (*n* = 4) mice were euthanized and peritoneal lavage cells were collected using 10 ml of PBS with 10% FBS. Peritoneal cells were spun down, resuspended in X-VIVO-10 (Lonza) containing 1% penicillin/streptomycin/glutamine, and 500,000 cells per well were seeded in non-tissue culture-treated 24-well plate and incubated at 37 °C and 5% carbon dioxide for 16 h. Nonadherent cells were then removed by extensive washing and adherent cells were stained with anti-CD11b (1:100, eBioscience #11-0112-82) and anti-F4/80 (1:100, BioLegend #123114) antibodies and analyzed by flow cytometry to verify peritoneal macrophage purity (>95%). Thymus was collected from a 3-weeks old mouse, strained through a 70 μm filter (Fisher) and thymocyte apoptosis was induced by incubation in 10μM dexamethasone (Calbiotech) in RPMI 1640 media containing 10% FBS and 1% penicillin/streptomycin/glutamine at 37 °C and 5% carbon dioxide for 4 h. Apoptosis was verified by Annexin V (BD Bioscience #550474) and 7AAD (Thermo Fisher) staining, following manufacturer’s instructions, and flow cytometry acquisition using FACS Diva and analysis using FlowJo (BD Bioscience). Apoptotic thymocytes were stained with 1 μM CypHer-5E (GE Healthcare) and added to peritoneal macrophages at a 10:1 (target:phagocyte) ratio for 30 min at 37 °C and 5% carbon dioxide. As a control, some macrophages were treated with cytochalasin D (Sigma, 1 μM) for 30 min prior to adding apoptotic thymocytes and throughout the efferocytosis assay to inhibit the apoptotic corpse uptake. Peritoneal macrophages were then washed extensively to remove unbound targets, stained with anti-CD11b (1:100, eBioscience #11-0112-82), and efferocytosis was analyzed by flow cytometry.

### CRISPR/Cas9-mediated gene deletion in Hoxb8 cells

Immortalized ER-Hoxb8 primary macrophage precursors (Hoxb8 cells) were generated as previously reported^46^ from mice with constitutive expression of Cas9 and GFP (Jackson Laboratories, stock number 026179). Macrophage precursor cells were maintained in α-MEM containing 100 ng/ml granulocyte-macrophage-colony-stimulating factor (GM-CSF) and 0.5 μM β-estradiol (Sigma) and differentiated into macrophages and osteoclasts as described above. LentiCas9-EGFP was a gift from P. Sharp and F. Zhang (Addgene plasmid 63592). Gene deletion in Hoxb8 cells was performed using lentiGuide-Puro sgRNA plasmid (gift from F. Zhang, Addgene plasmid 52963) and puromycin (5 μg/ml) selection. The deletion was verified by immunoblot analysis using the anti-ELMO1 (1:1000, in-house made rabbit polyclonal antibody)^5^, anti-Cathepsin G (1:1000, LS-C373215, LSBio) or anti-Myeloperoxidase (1:1000, AF3667, R&D Systems) antibodies.

Guide RNAs used were the following:

*Elmo1* guide, 5′- CACCGGATAGGCGGAGGTGCATCC-3′ and 3′-CCTATCCGCCTCCACGTAGGCAAA-5′.

*Ctsg* guide 5′- CACCGCTGGGTCCTTTCTCGCATT-3′ and 3′-CGACCCAGGAAAGAGCGTAACAAA-5′.

*Mpo* guide 5′-CACCGTCGTTGTAAGATCGGTACT-3′

3′-CAGCAACATTCTAGCCATGACAAA-5′.

*Aoah* guide, 5′-CACCGGCGTACATTTGTTCAGGAG-3′ and

3′-CCGCATGTAAACAAGTCCTCCAAA-5′.

*Rasgef1a* guide, 5′-CACCGCGGTCACCCGTTGTGGACAA-3′ and

3′-CGCCAGTGGGCAACACCTGTTCAAA-5′.

*Ero1lb* guide, 5′-CACCGGGTTCCGCCGGGCCGTTAC-3′ and

3′-CCCAAGGCGGCCCGGCAATGCAAA-5′

### Microscopy

Mature osteoclasts were fixed in ice-cold methanol or PBS containing 4% paraformaldehyde and stained with anti-vATPase subunit e1 (1:1000, PA5-29899, Thermo Fisher), anti-Cathepsin K (1:100, ab37259, Abcam), anti-Vinculin (1:100, V9264, Sigma), or control antibody (1:100, 5415 S, Cell Signaling), followed by fluorescently labeled secondary antibodies, following manufacturer’s instructions (Fisher). Some cells were also stained with Alexa Fluor 647-labeled Phalloidin (Thermo Fisher), following manufacturer’s instructions. The nuclei were stained with 1 µg/ml of Hoechst 33342 (Fisher) in PBS. Images were taken on an EVOS FL Auto (Thermo Fisher) and analyzed using the accompanying software. Because of intense TRAP staining in *Elmo1*^*−/−*^ osteoclast cultures, we counted the number of osteoclasts after cathepsin K staining by immunofluorescence. Cathepsin K-positive cells with three or more nuclei were counted using Image J. *RosaYFP* and Phalloidin stained osteoclast cultures were imaged on a Zeiss Imager Z2 with Apotome and analyzed using the AxioVision 4.8 software (Zeiss).

### Quantitative RT-PCR

Total RNA was isolated from cultured cells or pulverized paws using the RNA Easy kit (Qiagen) and cDNA prepared using the QuantiTect kit (Qiagen) according to manufacturer’s instructions. Quantitative gene expression for target and housekeeping genes was done using Taqman probes (Thermo Fisher Scientific, listed in Supplementary Table 4) run on a StepOnePlus Real Time PCR System (Applied Biosystems).

### Immunoblotting

Protein extracts were prepared from cultured cells or pulverized paws using RIPA lysis buffer with added protease inhibitors cocktail (Calbiochem), 1 mM phenylmethylsulfonyl fluoride (PMSF, Sigma) and 1 mM sodium orthovanadate (Sigma). Equal amounts of protein extract were loaded on TGX Precast gels (Bio-Rad), subjected to SDS-PAGE and transferred onto PVDF membranes using the Trans-Blot Turbo transfer system (Bio-Rad). In some experiments, active Rac1 was pulled down from equal amounts of protein extract using the GST-tagged Rac1 substrate CRIB^5^. Immunoblotting was performed using an in-house made anti-ELMO1 rabbit polyclonal antibody (1:1000, in-house made rabbit polyclonal antibody)^5^, anti-Rac1 (1:500, Cytoskeleton #ARC03), anti-ERK2 (1:3000, Santa Cruz #154-G), anti-GAPDH-HRP (1:10,000, Sigma #G9295), or anti-beta-Actin-HRP (1:100,000, Sigma #A3854). Blots were exposed using the Western Lightning Plus ECL kit (Perkin–Elmer) on the ChemiDoc Touch imaging system and analyzed using ImageLab (Bio-Rad).

### Proteomics and Mass Spectrometry

*Elmo1*^*+/+*^ and *Elmo1*^*−/−*^ osteoclasts were labeled with EZ-Link^TM^ NHS-Biotin (Thermo Fisher) following manufacturer’s instructions. Cell extracts were prepared in Triton X-100 lysis buffer (20 mM Tris, pH 8.0, 137 mM NaCl, 10% glycerol, 0.2% Triton X-100, protease inhibitors cocktail (Calbiochem), 1 mM PMSF, and 5 mM sodium orthovanadate) for 1 h on ice. Lysates were cleared by centrifugation for 10 min at 16,800x *g*. ELMO1 was immunoprecipitated using the mouse anti-ELMO1 antibody made in-house (clone #7G9-A10-A3-D7)^5^ and Protein A agarose beads (Amersham), and immune complexes were washed and split into two parts. One part was subjected to SDS-PAGE. The second part was heated for 5 min at 95 °C and the agarose beads were removed by centrifugation. Biotinilated proteins in the remaining immunoprecipitate were enriched by pulldown with NeutrAvidin^TM^ agarose (Pierce, following manufacturer’s instructions) and subjected to SDS-PAGE. The gels were stained with SimplyBlue Safe stain (Invitrogen) and destained with water. The gel bands were excised and frozen at −80 °C. After de-staining in 25 mM NH_4_HCO_3_ (pH 8.3) with 50% acetonitrile and drying in a vacuum centrifuge, the gel bands were digested with 100 μL of trypsin (Promega) (20 μg/mL) at 37 °C for 16 h. The digested peptides were extracted three times with 100 μL 70% acetonitrile, 5% formic acid in a sonication bath. Each peptide sample was dried in a vacuum centrifuge and resuspended in 16 μL 0.1% TFA. Each gel band digest was analyzed by nanoflow liquid chromatography (LC) (Easy-nLC1200, Thermo Fisher Scientific Inc.) coupled online with a an Orbitrap Fusion Lumos Tribrid MS (Thermo Fisher Scientific), samples were loaded on a C18 nano trap column, (Acclaim PepMap100 C18, 2 cm, nanoViper, Thermo Scientific) and resolved on a C18 Easy-Spray column (Acclaim PepMap RSLC C18, 2 μm, 100 Å, 75 μm x 500 mm, nanoViper, Thermo Scientific) with a linear gradient of 2% mobile phase B (95 % acetonitrile with 0.1% formic acid) to 32% mobile phase B within 60 min at a constant flow rate of 250 nL/min. The C18 Easy-Spray column was heated at 50 °C during the analysis. The 12 most intense molecular ions in each MS scan were sequentially selected for high-energy collisional dissociation (HCD) using a normalized collision energy of 35%. The mass spectra were acquired at the mass range of *m/z* 400–1600. The Easy-Spray ion source (Thermo Scientific) capillary voltage and temperature were set at 2.0 kV and 275 °C, respectively. Dynamic exclusion (15 s) was enabled during the MS/MS data acquisition to minimize redundant peptide fragmentation events. The RF lens was set to 30% during the MS analysis and both MS1 and MS2 spectra were collected in profile mode. Data were searched against a Swiss-Prot mouse protein database (http://www.uniprot.org/uniprot/) using Proteome Discoverer (v.2.2.0.388, Thermo Fisher Scientific) via Mascot (v. 2.6.0, Matrix Science Inc.) with the automatic decoy search option set followed by false-discovery rate (FDR) processing by Percolator. Data were searched with a precursor mass tolerance of 10 ppm and a fragment ion tolerance of 0.05 Da, a maximum of two tryptic miscleavages, and dynamic modifications for oxidation (15.9949 Da) on methionine residue and biotinylation (226.0776 Da) on lysine residues. The resulting peptide spectral matches (PSMs) were filtered using an FDR of ≤1% (Percolator *q* value ≤0.01). Protein networks were analyzed via STRING (www.string-db.org). Colored nodes indicate query proteins (colors were assigned arbitrarily by the program). Filled nodes represent proteins with known or predicted 3D structure. Known protein interactions are indicated by the magenta (experimentally determined) and cyan (curated database indicated) colored lines. Predicted protein interactions are indicated by green (gene neighborhood), olive (text-mining), black (co-expression), and violet (protein homology) colored lines.

### RNA-seq analysis

Osteoclasts were differentiated from the bone marrow of *Elmo1*^*+/–*^ or *Elmo1*^*−/−*^ mice with 50 ng/ml of GST-RANKL for 7 days, as described above. Total RNA was extracted, and an mRNA library was prepared using the Illumina TruSeq platform and followed by transcriptome sequencing using the NextSeq System Suite for an Illumina NextSeq 500 cartridge. Cultures from four mice per group, two mice per condition per experiment, were sequenced. The statistical software package R (version 3.3.2) was used for all analyses. The Bioconductor package DESeq2 was used for the normalization of RNA-seq data. Due to the inherent variability in osteoclast cultures, we analyzed the two groups of mice (two mice per condition) against each other to identify genes for further investigation as well as heatmaps. We used a statistical method previously established for highly variable TCR sequencing data^47^ that analyzes the average of the experimental condition divided by the sum of the control and experimental conditions. The resulting value is a ratio between 0 and 1 that provides a standardized metric for genes that are significantly up or down relative to the null value of 0.5. Heatmaps were created using the R package gplots via the heatmap.2 package. R code used for bioinformatics analysis and heatmap generation is available upon request.

### GWAS and experimental linkage analysis

SNP by disease and SNP by phenotype interactions were determined using GWASdb’s SNP-disease and SNP-phenotype association databases. The data are plotted using a standardized *p* value as reported^48^. For the determination of experimental linkage, a search of curated databases such as the DISEASES experimental gene-disease association database was performed (databases listed in Supplementary Table 1). The significance of the experimental linkage association was determined via a previously established aggregated score^49–53^.

### Statistical analysis

Statistical significance was determined using GraphPad Prism 5, 6, and 7 using unpaired Student’s two-tailed *t*-test or two-way ANOVA. The variance was similar between groups. No inclusion/exclusion criteria were pre-established. A *p* value of <0.05 (indicated by one asterisk), <0.01 (indicated by two asterisks), <0.001 (indicated by three asterisks), and <0.0001 (indicated by four asterisks) were considered significant.

### Illustration

For preparation of figures, we have modified illustrations available through www.motifolio.com.

### Reporting Summary

Further information on research design is available in the Nature Research Reporting Summary linked to this article.

## Supplementary information

Supplementary Information

Description of Additional Supplementary Files

Supplementary Movie 1

Reporting Summary

## Data Availability

The sequencing data generated in this study have been deposited in the GEO database under accession code “GSE164826”. The proteomics data generated in this study have been deposited in the ProteomeXchange under accession code “PXD023578”. All other relevant data supporting the key findings of this study are available within the article and its Supplementary Information files or from the corresponding author upon reasonable request. Source data are provided with this paper.

## Source data

Source Data

## Code availability

The data that support the findings from this study, including the R code used for bioinformatics analysis and heatmap generation, are available from the corresponding authors upon request.
