# Supplementary material for: ELMO1 signaling is a promoter of osteoclast function and bone loss
Source: Nat Commun. 2021 Aug 17;12:4974. doi: 10.1038/s41467-021-25239-6 (PMC8371122; doi:10.1038/s41467-021-25239-6)
Supplement: Supplementary file 1 — Supplementary Information [file 41467_2021_25239_MOESM1_ESM.pdf]

Supplementary Information

ELMO1 signaling is a promoter of osteoclast function and bone loss

Sanja Arandjelovic, Justin S.A. Perry, Ming Zhou, Adam Ceroi, Igor Smirnov, Scott F. Walk, Laura S. Shankman, Isabelle Cambré, Suna Onengut- Gumuscu, Dirk Elewaut, Thomas P. Conrads and Kodi S. Ravichandran

Supplementary Figure 1. Loss of ELMO1 decreases bone erosion in arthritis.

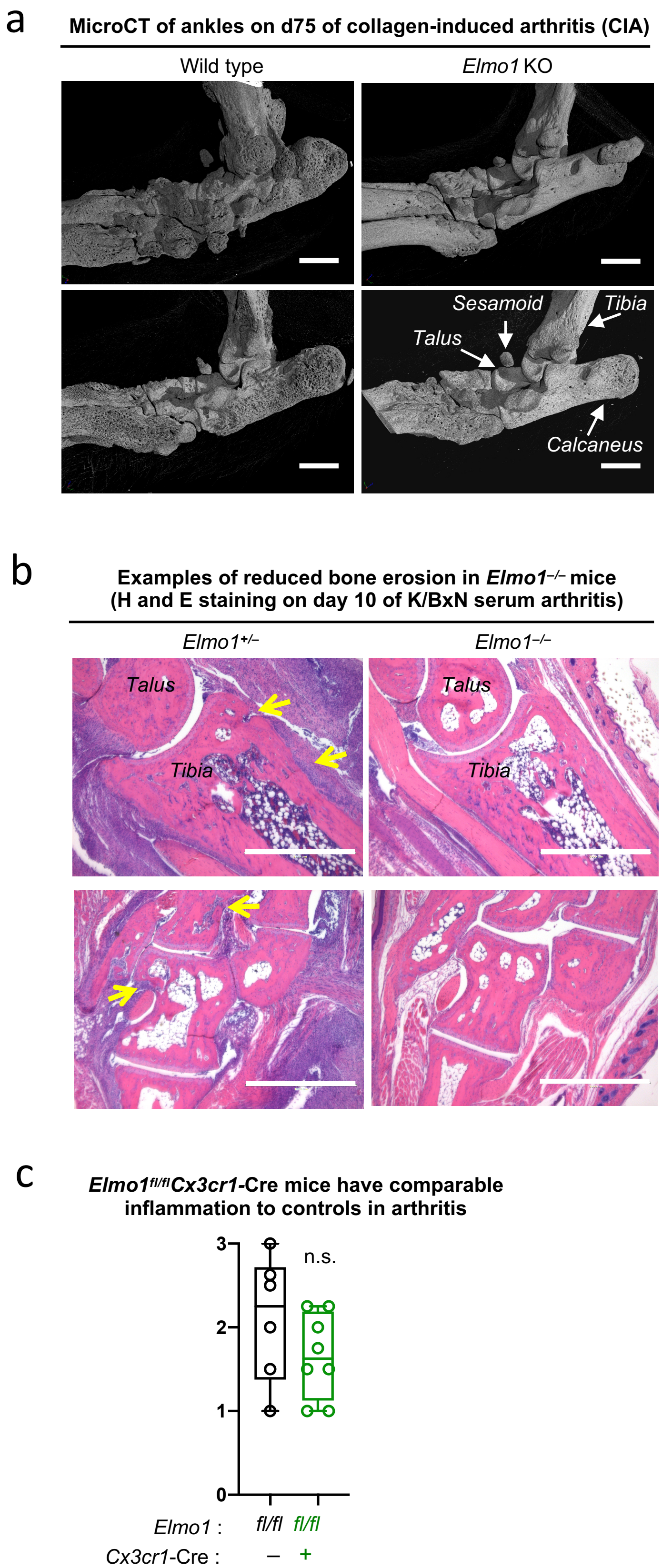

Supplementary Figure 1. Loss of ELMO1 decreases bone erosion in arthritis.

a) MicroCT images from ankles of *Elmo1*$^{+/+}$-DBA (wild type, n=3) and *Elmo1*$^{-/-}$-DBA (KO, n=3) mice on day 75 of collagen induced arthritis. Two mice per group are shown. Scale bar = 1 mm.

b) Representative sections of hind limb bone erosions in histology sections from *Elmo1*$^{+/-}$ (n=7) and *Elmo1*$^{-/-}$ (n=7) mice stained with hematoxylin and eosin (H and E) on day 10 after K/BxN serum injection. Examples of bone erosions are indicated with yellow arrows. Scale bar = 1 mm.

c) Inflammation was quantified in the H&E-stained histology sections of female *Elmo1*$^{fl/fl}$*Cx3cr1*-Cre (green, n=8) and *Elmo1*$^{fl/fl}$ (n=6) on day 10 of K/BxN serum induced arthritis. Each symbol represents an individual animal. Data are presented in a Box and Whiskers format with box indicating 25th to 75th percentile, all data points shown, and median indicated. Source data are provided as the Source data file.

Supplementary Figure 2. Loss of ELMO1 does not reduce osteoclast numbers or osteoblast mineralization.

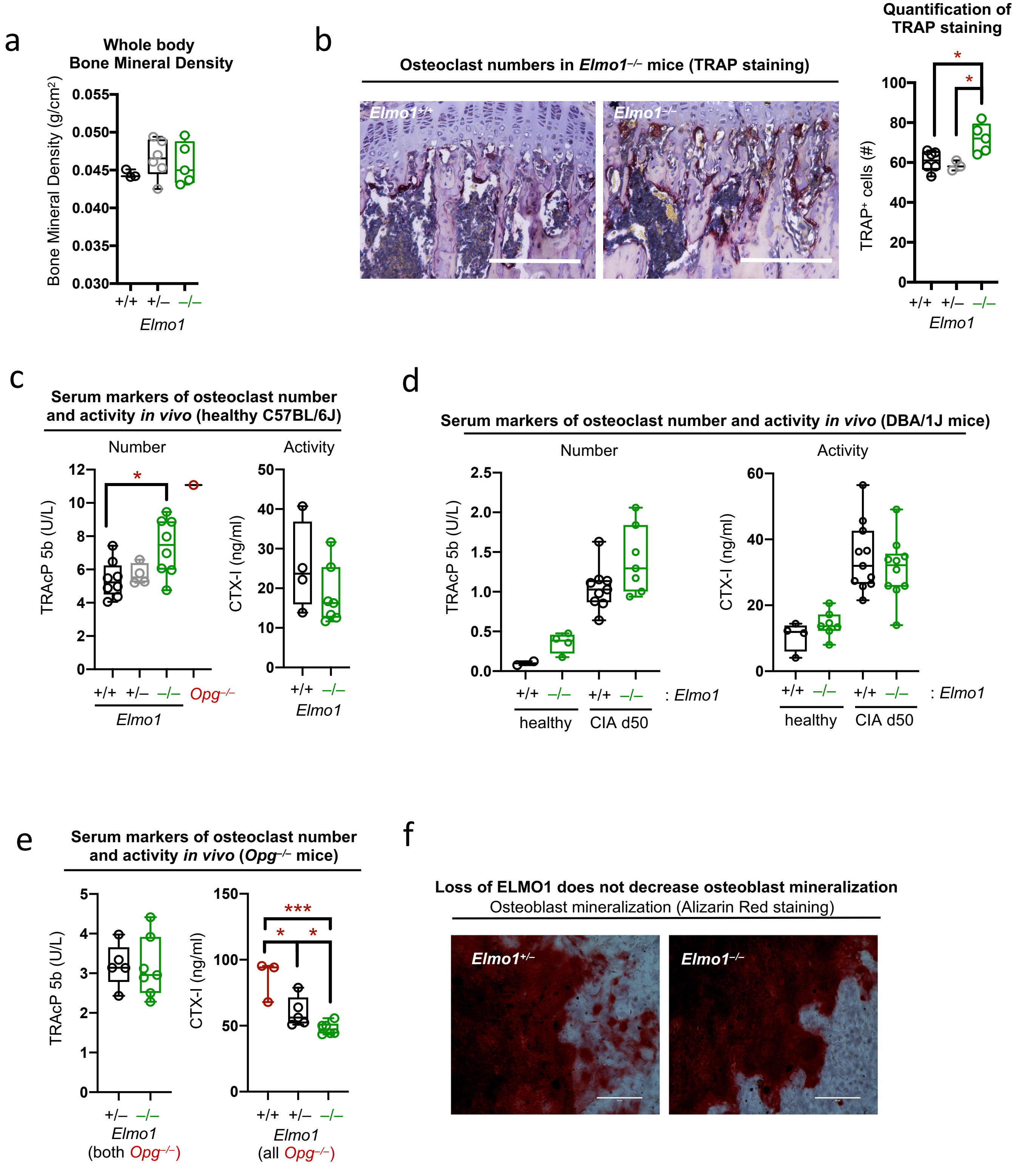

Supplementary Figure 2. Loss of ELMO1 does not reduce osteoclast numbers or osteoblast mineralization.

a) Whole body mineral density was analyzed by DEXA scan in 16 week old *Elmo1*$^{+/+}$ (black, n=3), *Elmo1*$^{+/-}$ (grey, n=6) and *Elmo1*$^{-/-}$ (green, n=5) mice. Each symbol represents a mouse. Data are presented in a Box and Whiskers format with box indicating 25th to 75th percentile, all data points shown and median indicated.

b) Osteoclast numbers in tibias from *Elmo1*$^{+/+}$ (black, n=6), *Elmo1*$^{+/-}$ (grey, n=3) and *Elmo1*$^{-/-}$ (green, n=5) mice were quantified in the 1 mm area directly below the bone growth plate in tibial bones (representative images shown). Each symbol represents a mouse. Data are presented in a Box and Whiskers format with box indicating 25th to 75th percentile, all data points shown and median indicated. *p<0.05, Student's t-test, two-tailed, unpaired. Scale bar = 0.4 mm. *Elmo1*$^{-/-}$ mice likely have a compensatory increase in osteoclast numbers (as seen with defective osteoclast function).

c) Osteoclast number and activity were evaluated by analysis of serum levels of TRAcP 5b and CTX-I, respectively (see Methods), in healthy one year-old *Elmo1*$^{+/+}$ (black, n=8 for TRAcP 5b, n=4 for CTX-I), *Elmo1*$^{+/-}$ (grey, n=4) and *Elmo1*$^{-/-}$ (green, n=8 for TRAcP 5b, n=7 for CTX-I) female mice. Each symbol represents a mouse. One age- and sex-matched *Opg*$^{-/-}$ (red) mouse is shown as a reference point for increased osteoclast numbers. Data are presented in a Box and Whiskers format with box indicating 25th to 75th percentile, all data points shown and median indicated. *p<0.05, Student's t-test, two-tailed, unpaired.

d) Serum TRAcP 5b levels in *Elmo1*$^{+/+}$DBA (black, n=2 healthy, 9 CIA) and *Elmo1*$^{-/-}$DBA (green, n=4 healthy, 7 CIA) and CTX-I levels in *Elmo1*$^{+/+}$DBA (black, n=4 healthy, 11 CIA) and *Elmo1*$^{-/-}$DBA (green, n=7 healthy, 10 CIA) male mice show increased osteoclast numbers and activity in arthritic mice (day 50 of collagen-induced arthritis), compared to healthy controls. Each symbol represents a mouse. Data are presented in a Box and Whiskers format with box indicating 25th to 75th percentile, all data points shown and median indicated.

e) Serum TRAcP 5b and CTX-I levels in one year-old male *Opg*$^{-/-}$*Elmo1*$^{+/+}$ (red, n=3), *Opg*$^{-/-}$*Elmo1*$^{+/-}$ (black, n=5) and *Opg*$^{-/-}$*Elmo1*$^{-/-}$ (green, n=7) mice indicate an ELMO1 dose-dependent decrease in osteoclast activity. Each symbol represents a mouse. Data are presented in a Box and Whiskers

format with box indicating 25th to 75th percentile, all data points shown and median indicated. *$p<0.05$, ***$p<0.001$, Student's t-test, two-tailed, unpaired.

f) Alizarin Red staining of mineralized matrix deposition indicates comparable function of *Elmo1*$^{+/-}$ and *Elmo1*$^{-/-}$ osteoblasts differentiated from the bone marrow. Scale bar = 0.2 mm. Representative of three independent experiments. Source data are provided as the Source data file.

Supplementary Figure 3. Loss of ELMO1 in Hoxb8 cells reduces osteoclast function.

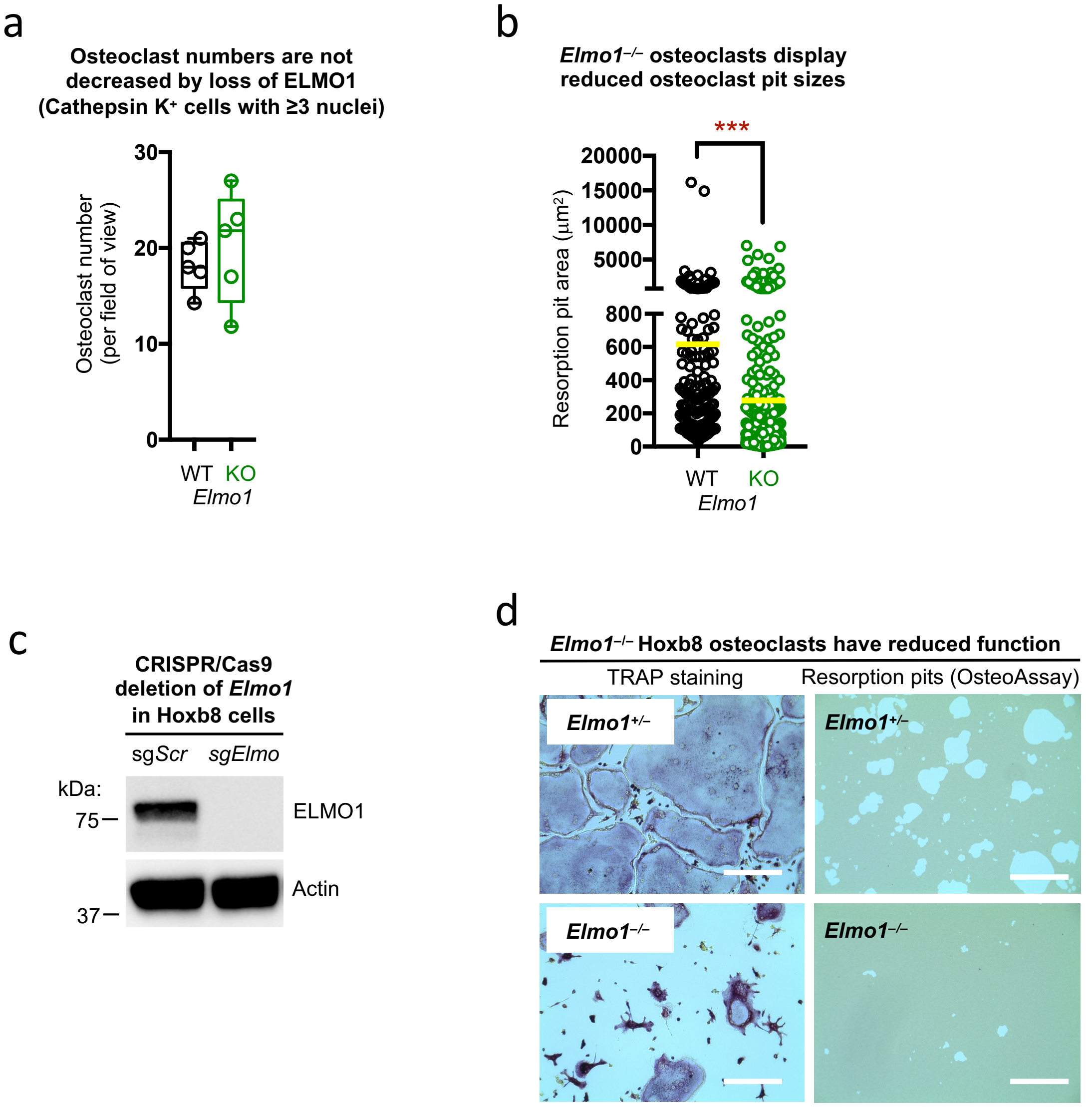

Supplementary Figure 3. Loss of ELMO1 in Hoxb8 cells reduces osteoclast function.

a) Comparable number of *ex vivo* mature osteoclasts with >3 nuclei are derived from *Elmo1*$^{-/-}$ (green, n=4) mice, compared to WT (black, n=5). Cathepsin K^{+} cells with 3 or more nuclei per field in WT and *Elmo1*$^{-/-}$ (KO) osteoclast cultures on day 7 of differentiation. Each symbol represents culture from an individual animal. Data are presented in a Box and Whiskers format with box indicating 25th to 75th percentile, all data points shown and median indicated.

b) Quantification of resorption pit areas of *Elmo1*$^{+/+}$ (black) and *Elmo1*$^{-/-}$ (green) osteoclasts on OsteoAssay plates, as in Figure 3d, with individual measurements shown. Data are presented in a Scatter dot plot format with the average pit size (mean) depicted by the yellow line. Data compiled from 4 WT and 8 KO mice are shown. ***$p<0.0006$, Student's t-test, two-tailed, unpaired.

c) ELMO1 protein expression in Cas9 expressing Hoxb8 cells carrying an *Elmo1* targeting guide RNA (sg*Elmo1*) or a scrambled guide RNA (sg*Scr*). Actin protein expression was used as loading control. Representative of three independent experiments is shown.

d) Hoxb8 cells established from *Elmo1*$^{+/-}$ and *Elmo1*$^{-/-}$ mice were differentiated into osteoclasts on OsteoAssay plates. On day 7 of differentiation, osteoclasts were TRAP stained (left panels) or removed to reveal resorption pits (right panels). Scale bar = 0.2 mm. Representative of three independent experiments is shown. Source data are provided as the Source data file.

Supplementary Figure 4. Growth and differentiation of *Elmo1* KO osteoclasts are not impaired.

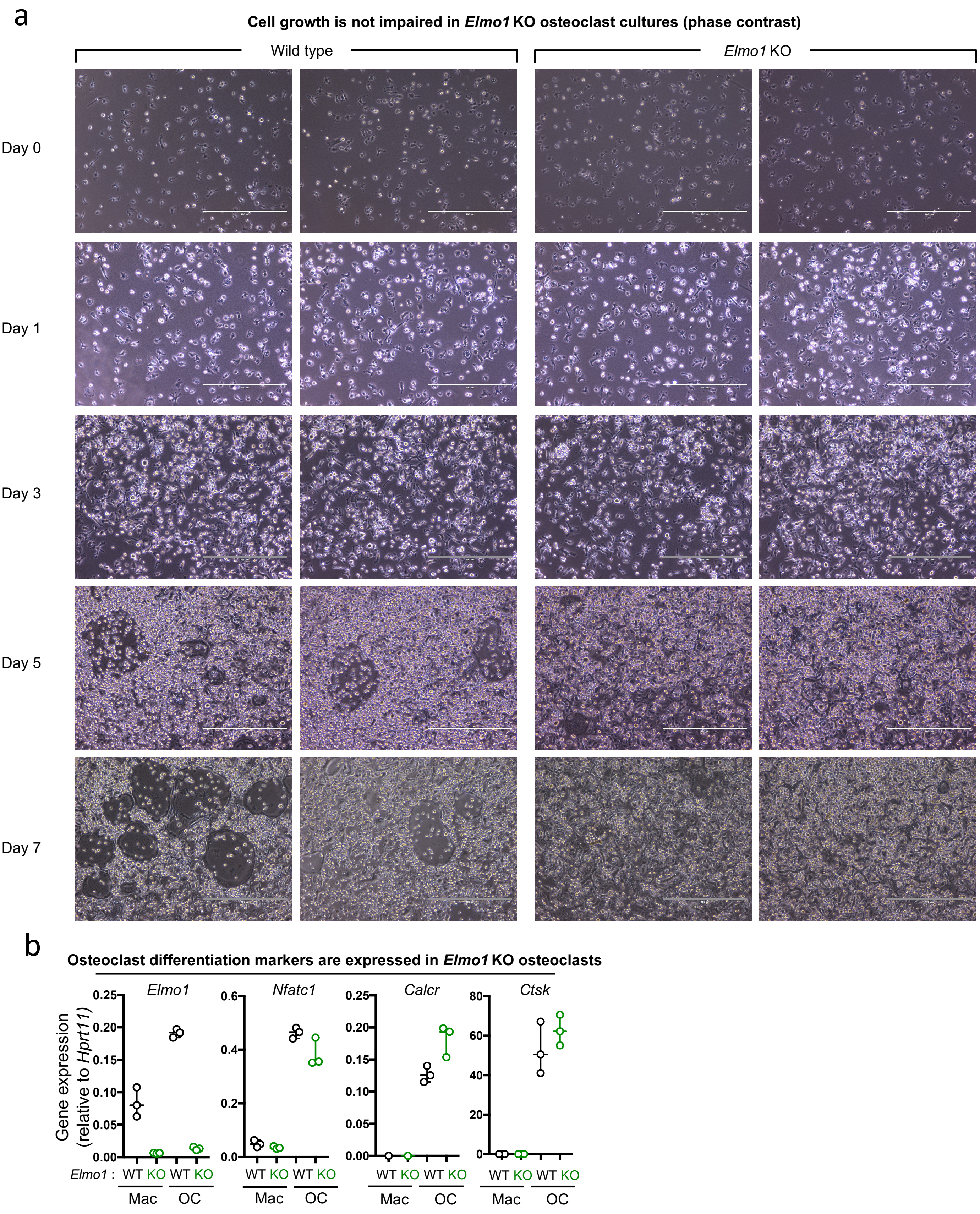

Supplementary Figure 4. Growth and differentiation of *Elmo1* KO osteoclasts is not impaired.

a) Bone marrow from two *Elmo1*$^{+/+}$ (wild type) and two *Elmo1*$^{-/-}$ (KO) mice was differentiated into osteoclasts on OsteoAssay plates and phase contrast pictures were taken daily. Scale bar = 0.4mm.

b) Normal maturation of *Elmo1*$^{-/-}$ osteoclasts. Expression of osteoclast maturation genes (along with *Elmo1*) was analyzed by quantitative RT-PCR in macrophages (control) and day 7 osteoclasts differentiated from the bone marrow of *Elmo1*$^{+/+}$ (WT, black) and *Elmo1*$^{-/-}$ (KO, green) mice. Each symbol represents culture from an individual animal. Data are presented in a Box and Whiskers format with all data points shown and median indicated. Representative of three independent experiments is shown. Source data are provided as the Source data file.

Supplementary Figure 5. Loss of ELMO1 does not decrease macrophage efferocytosis or osteoclast integrin expression.

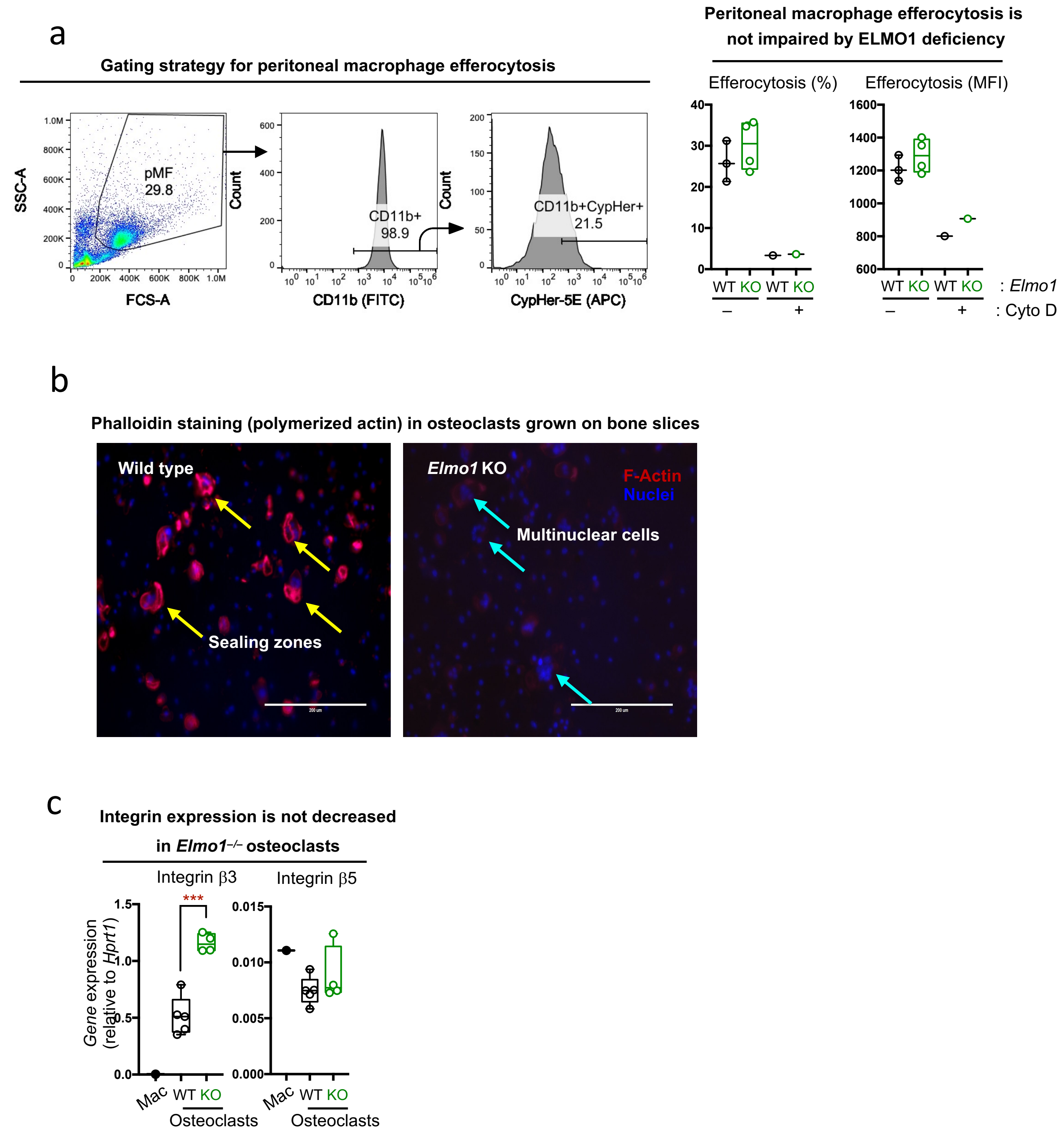

Supplementary Figure 5. Loss of ELMO1 does not decrease macrophage efferocytosis of osteoclast integrin expression.

a) Macrophages from *Elmo1*$^{-/-}$ mice display normal efferocytosis of apoptotic cells. Resident peritoneal macrophages from *Elmo1*$^{+/+}$ (WT, black) and *Elmo1*$^{-/-}$ (KO, green) mice were incubated with fluorescently labeled apoptotic thymocytes *ex vivo* for 30min in the presence or absence of cytochalasin D (CytoD), as described in the Methods. Percent of macrophages (%) with engulfed apoptotic thymocytes and the mean fluorescent index (MFI) were quantified by flow cytometry. Previous studies have shown that compensatory expression of *Elmo2* in macrophages can overcome *Elmo1* deficiency with respect to efferocytosis. Data are presented in a Box and Whiskers format with box indicating 25th to 75th percentile, all data points shown and median indicated. Representative of two independent experiments is shown.

b) Phalloidin (F-actin, red) and Hoechst (nuclei, blue) staining (pseudo-colored) of *Elmo1*$^{+/+}$ (wild type, n=2) and *Elmo1*$^{-/-}$ (KO, n=2) osteoclasts grown on bovine bone slices. Yellow arrows indicate osteoclast sealing zones/rings. Cyan arrows indicate multinuclear cells. Scale bar = 0.2 mm. Representative of two independent experiments is shown.

c) Normal expression of genes encoding integrin β3 and integrin β5 in *Elmo1*$^{-/-}$ osteoclasts. Gene expression was analyzed by quantitative RT-PCR in macrophages (BMDM) and day 7 osteoclasts differentiated from the bone marrow of *Elmo1*$^{+/+}$ (WT, black) and *Elmo1*$^{-/-}$ (KO, green) mice. Data are presented in a Box and Whiskers format with box indicating 25th to 75th percentile, all data points shown and median indicated. ***$p<0.001$, Student's t-test, two-tailed, unpaired. Each symbol represents culture from an individual animal. Source data are provided as the Source data file.

Supplementary Figure 6. *Elmo1* regulated genes influence osteoclast function.

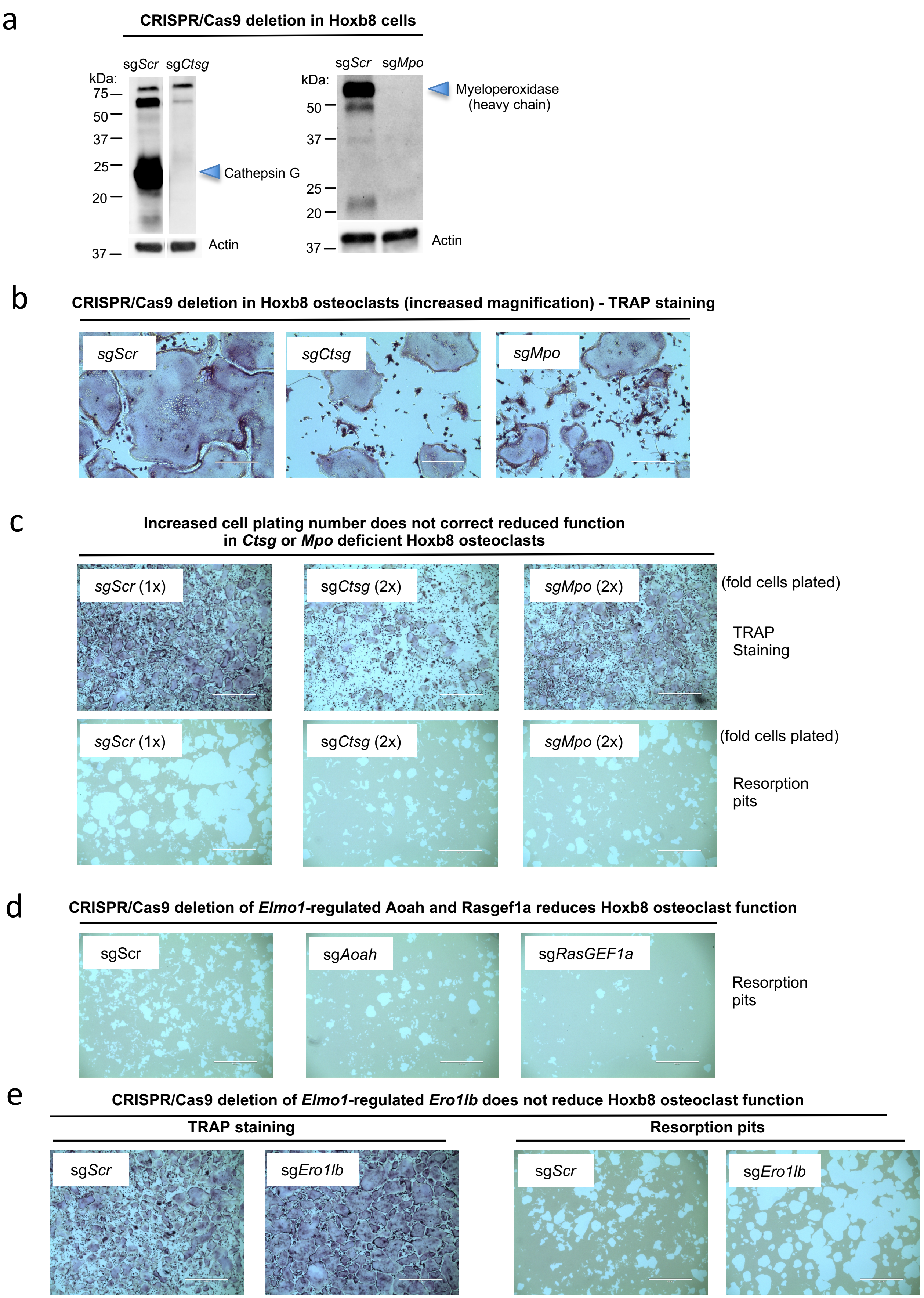

Supplementary Figure 6. ***Elmo1*** **regulated genes influence osteoclast function.**

a) Cas9 expressing Hoxb8 macrophage precursor cells were transduced with guide RNA (sg) targeting cathepsin G (*Ctsg*) or myeloperoxidase (*Mpo*). Loss of targeted protein was verified in whole cell extracts by immunoblotting. The Ctsg lanes are from the same blot but not run next to each other. In the right panel, the MPO heavy chain is indicated, and the lower bands likely represent MPO degradation products, as this was again lost in the cells with *Mpo* deletion. Actin was used for loading control. Representative of two experiments is shown.

b) TRAP staining of Cas9-Hoxb8 osteoclasts stably expressing indicated guide RNA (sg), differentiated for 7 days on OsteoAssay plates. Scale bar = 0.2 mm. Refers to data shown in main Figure 3c. Representative of more than three independent experiments is shown.

c) Increased cell plating numbers does not correct reduced function of cathepsin G or myeloperoxidase-deficient osteoclasts. TRAP staining (top panels) and resorption pits (bottom panels) of Cas9-Hoxb8 osteoclasts stably expressing indicated guide RNA (sg) targeting *Ctsg* or *Mpo*, and differentiated for 7 days on OsteoAssay plates. Scale bar = 1 mm. One experiment is shown.

d) Resorption pits of Cas9-Hoxb8 osteoclasts stably expressing indicated guide RNAs targeting acyloxyacyl hydrolase (*Aoah*) and RasGEF1, differentiated for 7 days on OsteoAssay plates. Scale bar = 1 mm. Representative of two independent experiments is shown.

e) Not all enzymes targeted in Hoxb8 osteoclasts decrease bone resorption, as targeting endoplasmic reticulum oxidoreductase 1 beta (*Ero1lb*) increases osteoclast activity. TRAP staining (left panels) and resorption pits (right panels) of Cas9-Hoxb8 osteoclasts stably expressing sg*Ero1lb* differentiated for 7 days on OsteoAssay plates. Scale bar = 1 mm. One experiment is shown. Source data are provided as the Source data file.

Supplementary Figure 7. ELMO1 inhibition impairs osteoclast function.

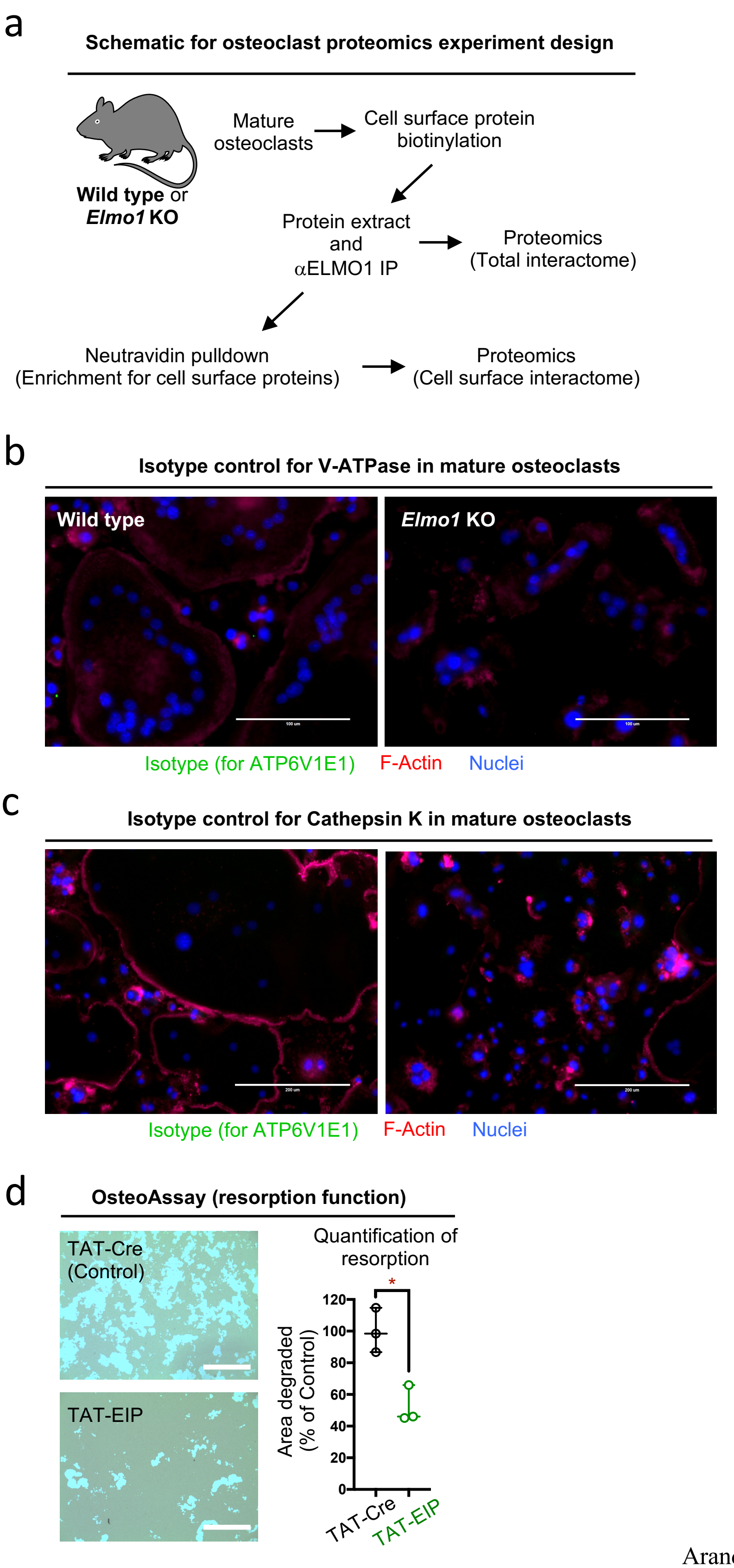

Supplementary Figure 7. ELMO1 inhibition impairs osteoclast function.

a) Schematic of the osteoclast preparation steps to identify the ELMO1 protein interactome via proteomics.

b,c) Bone marrow from *Elmo1*$^{+/+}$ (wild type) and *Elmo1*$^{-/-}$ (KO) mice was differentiated into osteoclasts and the cells stained as indicated. Scale bar = 0.1 mm. Representative of two independent experiments is shown.

d) Wild type osteoclasts were differentiated on OsteoAssay plates. From day 3, 10µM TAT-EIP (green) or TAT-Cre (black) were added and changed daily until day 7, when the cells were removed to reveal and quantify resorption pits. Scale bar = 0.4mm. Data are presented in a Box and Whiskers format with all data points shown and median indicated. *p=0.0108, Student's t-test, two-tailed, unpaired. Data from cultures prepared from three animals is shown. Source data are provided as the Source data file.

Supplementary Table 1. Details of databases used and specific SNPs associated with the indicated genes.

Data Aggregation

Database Name	DOI
GWASdb	10.1093/nar/gkr1182
NHGRI GWAS Catalog	10.1093/nar/gkt1229
Genetic Association Database	10.1038/ng0504-431
GEO Signatures of Differentially Expressed Genes for Diseases	10.1093/nar/gks1193
dbGAP: Database of Genotypes and Phenotypes	10.1093/nar/gkt1211
CTD Gene-Disease Associations	10.1093/nar/gku935
DISEASES Experimental Gene-Disease Assocation Evidence Scores	10.1016/j.ymeth.2014.11.020

SNPs

Gene	Chr	Position	SNP ID	PubMed ID	adj P-value	Population	Trait	SNP Type
Elmo1	7	37436854	rs11984075	21383967	5.00E-08	European(38053)	Celiac & RA	Intron
Elmo1	7	37427351	rs75351767	23143596	2.94E-07	European(27345)	RA	Intron
Elmo1	7	37427351	rs75351767	23143596	3.85E-05	European(27345)	RA (CCP positive)	Intron
Elmo1	7	37427351	rs75351767	23143596	5.95E-05	European(27345)	RA (CCP negative)	Intron
Elmo1	7	37067395	rs10488029	23382691	4.00E-06	European(2247)	IgG glycosylation	Intron
Rac1	7	6434904	rs836472	21452313	1.28E-04	East Asian (1558)	RA	Exon 4
Dock2	5	169461547	rs3763048	23382691	9.00E-06	European(2247)	IgG glycosylation	Intron
Mtus2	13	30140981	rs6490411	17982456	2.00E-07	NOPOP(397), Framingham(1211)	RA (CCP positive)	Intron
Mtus2	13	29786977	rs6490370	20436471	2.34E-05	European(1693)	Paget`s disease	Intron
Tnfsf10	3	23201811	rs20575	22480748	3.80E-02	European[154]	RA, αTNF Tx Response	Exon 4
Tnfsf10	3	23201811	rs20575	22480748	4.00E-02	European[154]	RA, αTNF Tx Response	Exon 4
Tnfsf10	3	23201811	rs20575	22480748	4.00E-02	European[154]	RA, αTNF Tx Response	Exon 4
Tjp1	15	30071279	rs12916064	23233654	4.07E-04	European(882)	RA, αTNF Tx Response	Intron
Tjp1	15	30008279	rs4780257	23233654	4.67E-04	European(882)	RA, αTNF Tx Response	Intron
Tjp1	15	30162502	rs12592592	23233654	9.97E-04	European(882)	RA, αTNF Tx Response	Intron
Cxcl10	4	76021147	rs8878	25702175	3.40E-02	European[780]	RA	Exon 4
Ifi44	1	79176868	rs4650376	23382691	2.00E-06	European(2247)	IgG Glycosylation	Intron
Ifi44	1	78987302	rs672561	23382691	9.00E-06	European(2247)	IgG Glycosylation	Intron
Mx2	21	42793791	rs459482	23382691	3.00E-07	European(2247)	IgG glycosylation	UTR-5
Mx2	21	42793791	rs459482	23382691	2.00E-06	European(2247)	IgG glycosylation	UTR-5
Exoc3l4	14	103485652	rs1106679	17982456	2.29E-05	NOPOP(397), Framingham(1211)	RA (CCP positive)	intron
Gpr137b	1	236335567	rs6702348	21452313	2.99E-05	Korean(1558)	RA	Intron
Gpr137b	1	236329513	rs7537965	21452313	2.68E-04	Korean(1558)	RA	Intron
Mpo	17	56313904	rs8067912	23382691	5.00E-07	European(2247)	IgG glycosylation	Intron
Mpo	17	56313904	rs8067912	23382691	1.00E-06	European(2247)	IgG glycosylation	Intron
Rgs11	16	375782	rs9921222	22504420	1.00E-16	East Asian, European(32961)	Bone mineral density	intron
Rgs11	16	375782	rs9921222	24945404	7.95E-05	NOPOP(588), Turkish(300), Moroccan(232), European(7976), Surinamese(289)	Bone mineral density	intron
Rgs11	16	375782	rs9921222	24945404	1.13E-04	NOPOP(588), Turkish(300), Moroccan(232), European(7976), Surinamese(289)	Bone mineral density	intron

Supplementary Table 2. *Elmo1* regulates transcriptional programs in osteoclasts.

Osteoclast-specific genes regulated by Elmo1

Symbol	Average Ratio	Symbol	Average Ratio	Symbol	Average Ratio	Symbol	Average Ratio
Mtus2	0.702472548	*Ifit1*	0.573271804	*Akr7a5*	0.537867345	*Nnmt*	0.396142394
Veph1	0.685436848	*Cmpk2*	0.572107827	*Cpne2*	0.537844345	*Ddit4l*	0.396223681
Tnfsf10	0.6948456	*Ppm1n*	0.570701647	*Dcakd*	0.537158692	*Pacrg*	0.396611649
Tjp1	0.668220745	*Oasl1*	0.567841645	*Parp12*	0.535007379	*Rac3*	0.398052676
Ifi203	0.659139732	*Gpr84*	0.566663339	*AW112010*	0.534012736	*Stard13*	0.400190071
Cxcl10	0.656263398	*Fam83f*	0.566465144	*Fbl*	0.53266786	*Nd1*	0.401865869
Gbp5	0.624309929	*Casp2*	0.566188877	*Zfp574*	0.532659004	*Nd6*	0.406516379
Ifit2	0.6104783	*Irgm2*	0.565579639	*Pde7a*	0.531195206	*Ank1*	0.414184164
Nxpe3	0.60880761	*Dtx3l*	0.561948371	*Sf3b4*	0.530235226	*Tead1*	0.415436963
Iigp1	0.607461161	*Ddx60*	0.561306911	*Slc25a22*	0.529426194	*Spp1*	0.416263299
Ifi44	0.602233924	*Oas3*	0.560526562	*Gpr137b-ps*	0.033089824	*Abca8b*	0.420248141
Timp3	0.598585693	*Pyhin1*	0.560443919	*Elmo1*	0.154205884	*Aldh1l2*	0.420269223
Mx2	0.594413765	*Hp*	0.559701536	*Sugct*	0.20832173	*Gfra1*	0.433384714
Exoc3l	0.592129987	*C1qtnf6*	0.559347576	*Ctsg*	0.277804087	*Rftn2*	0.439344583
Ifit3b	0.591880274	*Mybl2*	0.557512328	*Lce1g*	0.284926112	*Insl6*	0.439474543
Mnda	0.589232625	*Trim30d*	0.55628856	*Mpo*	0.291785946	*Crem*	0.440529212
Cxcl13	0.588579858	*Rab4a*	0.555364077	*Mgam*	0.298858881	*Stac2*	0.44054668
Ggt5	0.58821174	*Homez*	0.554876406	*Slc2a4rg*	0.309887733	*Stfa3*	0.441568128
Olfr56	0.586757353	*Tmem37*	0.548359973	*Dusp13*	0.32210814	*Mplkip*	0.446505442
Vcam1	0.586556136	*Parp11*	0.546538404	*Rgs11*	0.330432885	*Cnn2*	0.450597651
Gbgt1	0.580937203	*Herc6*	0.545880611	*Prtn3*	0.331496194	*Plk2*	0.451700265
Rsad2	0.580459627	*Il18bp*	0.545564638	*Sphk1*	0.356938064	*Eno3*	0.454929694
Ly6i	0.5799029	*Phf11d*	0.542957619	*Lrrc1*	0.365852943	*Sec11c*	0.45860763
Slfn5	0.577206583	*Trp53*	0.540782958	*Rasgef1a*	0.370780206	*Pgam1*	0.472816384
Slfn9	0.575337815	*Stat1*	0.540565898	*Aoah*	0.383732091	*Lyst*	0.478624137
Rmi2	0.574389708	*Cdt1*	0.538738455	*Hist1h1c*	0.385801037		
Ifit3	0.573517895	*Csad*	0.538222383	*Ero1lb*	0.394727865		

Supplementary Table 3 - String annotations for Figure 4

ELMO1 Cell Surface Protein Interactome

#node1	node2	node1_string_id	node2_string_id	neighborhood_on_chromosom	gene_fusion	phylogenetic_cooccurrenc	homology	coexpressio	experimentally_determined_interacti	database_annotatec	automated_textmining	combined_score
Atp6v1b2	Atp6v1e1	10090.ENSMUSP00000006435	10090.ENSMUSP0000001935	0.164	0	0	0	0.542	0.738	0.9	0.669	0.996
Cd44	Gpnmb	10090.ENSMUSP00000005218	10090.ENSMUSP0000003184	0	0	0	0	0.123	0	0	0.513	0.555
Cd44	Itgb1	10090.ENSMUSP00000005218	10090.ENSMUSP0000008745	0	0	0	0	0.102	0.155	0	0.87	0.892
Cd44	Sirpa	10090.ENSMUSP00000005218	10090.ENSMUSP0000009949	0	0	0	0	0.061	0	0.9	0.297	0.928
Eno1	Lcp1	10090.ENSMUSP00000079727	10090.ENSMUSP0000011627	0	0	0	0	0.049	0.263	0	0.245	0.425
Gpnmb	Itgb1	10090.ENSMUSP00000031840	10090.ENSMUSP0000008745	0	0	0	0	0.061	0	0	0.455	0.466
Itgb1	Itgb7	10090.ENSMUSP00000087457	10090.ENSMUSP0000000132	0	0	0	0.921	0.061	0	0.72	0.452	0.735
Itgb1	Lrp1	10090.ENSMUSP00000087457	10090.ENSMUSP0000004400	0	0	0	0	0	0	0	0.503	0.503
Itgb1	Itgb5	10090.ENSMUSP00000087457	10090.ENSMUSP0000006941	0	0	0	0.906	0.089	0	0.72	0.635	0.749
Itgb5	Itgb7	10090.ENSMUSP00000069416	10090.ENSMUSP0000000132	0	0	0	0.865	0	0	0.72	0.295	0.73
Lrp1	Sirpa	10090.ENSMUSP00000044004	10090.ENSMUSP0000009949	0	0	0	0	0.127	0	0	0.433	0.484

ELMO1 Total Protein Interactome

#node1	node2	node1_string_id	node2_string_id	neighborhood_on_chromosom	gene_fusion	phylogenetic_cooccurrenc	homology	coexpressio	experimentally_determined_interacti	database_annotatec	automated_textmining	combined_score
Atp12a	Atp4a	10090.ENSMUSP00000007340	10090.ENSMUSP0000000569	0	0	0.448	0.969	0	0	0.8	0.746	0.807
Atp12a	Atp6v0d1	10090.ENSMUSP00000007340	10090.ENSMUSP0000001330	0	0	0	0	0	0.217	0	0.375	0.49
Atp12a	Atp6v1c1	10090.ENSMUSP00000007340	10090.ENSMUSP0000002290	0	0	0	0	0.062	0.094	0	0.499	0.537
Atp12a	Atp6v1a	10090.ENSMUSP00000007340	10090.ENSMUSP0000011031	0.043	0	0	0	0.052	0.343	0	0.436	0.618
Atp12a	Atp6v0d2	10090.ENSMUSP00000007340	10090.ENSMUSP0000002990	0	0	0	0	0.06	0.217	0	0.899	0.919
Atp1a1	Atp6v0d1	10090.ENSMUSP00000039657	10090.ENSMUSP0000001330	0	0	0	0	0.061	0.329	0	0.206	0.456
Atp1a1	Atp2b1	10090.ENSMUSP00000039657	10090.ENSMUSP0000002010	0	0	0.446	0.652	0.099	0.544	0	0.351	0.675
Atp1a1	Atp6v1a	10090.ENSMUSP00000039657	10090.ENSMUSP0000011031	0.043	0	0	0	0.06	0.449	0	0.226	0.565
Atp1a1	Atp1a3	10090.ENSMUSP00000039657	10090.ENSMUSP0000009992	0	0	0.449	0.983	0	0	0.8	0.71	0.803
Atp1a1	Atp1a2	10090.ENSMUSP00000039657	10090.ENSMUSP0000008307	0	0	0.449	0.984	0	0	0.8	0.722	0.803
Atp1a1	Atp1a4	10090.ENSMUSP00000039657	10090.ENSMUSP0000010687	0	0	0.449	0.979	0	0	0.8	0.469	0.803
Atp1a2	Atp6v0d1	10090.ENSMUSP00000083077	10090.ENSMUSP0000001330	0	0	0	0	0	0.655	0	0.128	0.686
Atp1a2	Atp2b1	10090.ENSMUSP00000083077	10090.ENSMUSP0000002010	0	0	0.446	0.64	0.099	0.449	0	0.281	0.6
Atp1a2	Atp6v1a	10090.ENSMUSP00000083077	10090.ENSMUSP0000011031	0.043	0	0	0	0.054	0.68	0	0.193	0.735
Atp1a2	Atp1a4	10090.ENSMUSP00000083077	10090.ENSMUSP0000010687	0	0	0.449	0.98	0	0	0.8	0.701	0.804
Atp1a2	Atp1a3	10090.ENSMUSP00000083077	10090.ENSMUSP0000009992	0	0	0.449	0.983	0.071	0	0.8	0.713	0.809
Atp1a3	Atp2b1	10090.ENSMUSP00000099922	10090.ENSMUSP0000002010	0	0	0.446	0.642	0.139	0.203	0	0.19	0.426
Atp1a3	Atp6v1a	10090.ENSMUSP00000099922	10090.ENSMUSP0000011031	0.043	0	0	0	0.106	0.415	0	0.113	0.497
Atp1a3	Atp1a4	10090.ENSMUSP00000099922	10090.ENSMUSP0000010687	0	0	0.449	0.978	0	0	0.8	0.491	0.803
Atp1a4	Atp6v0d1	10090.ENSMUSP00000106874	10090.ENSMUSP0000001330	0	0	0	0	0	0.217	0	0.321	0.446
Atp2b1	Atp6v1c1	10090.ENSMUSP00000020107	10090.ENSMUSP0000002290	0	0	0	0	0.075	0.094	0	0.353	0.41
Atp2b1	Atp6v1a	10090.ENSMUSP00000020107	10090.ENSMUSP0000011031	0.043	0	0	0	0.132	0.167	0	0.275	0.431
Atp4a	Atp6v0d1	10090.ENSMUSP00000005692	10090.ENSMUSP0000001330	0	0	0	0	0	0.217	0	0.402	0.512
Atp4a	Atp6v1c1	10090.ENSMUSP00000005692	10090.ENSMUSP0000002290	0	0	0	0	0.062	0.094	0	0.499	0.537
Atp4a	Atp6v1a	10090.ENSMUSP00000005692	10090.ENSMUSP0000011031	0.043	0	0	0	0.052	0.343	0	0.436	0.618
Atp4a	Atp6v0d2	10090.ENSMUSP00000005692	10090.ENSMUSP0000002990	0	0	0	0	0	0.217	0	0.899	0.918
Atp6v0d1	Atp6v0d2	10090.ENSMUSP00000013304	10090.ENSMUSP0000002990	0	0	0	0.972	0	0.688	0.9	0.65	0.968
Atp6v0d1	Atp6v1c2	10090.ENSMUSP00000013304	10090.ENSMUSP0000002088	0	0	0	0	0.466	0.632	0.9	0.611	0.991
Atp6v0d1	Atp6v1c1	10090.ENSMUSP00000013304	10090.ENSMUSP0000002290	0	0	0	0	0.54	0.632	0.9	0.567	0.991
Atp6v0d1	Atp6v1a	10090.ENSMUSP00000013304	10090.ENSMUSP0000011031	0.172	0	0	0	0.364	0.609	0.9	0.746	0.993
Atp6v0d2	Atp6v1c2	10090.ENSMUSP00000029900	10090.ENSMUSP0000002088	0	0	0	0	0.466	0.632	0.9	0.697	0.993
Atp6v0d2	Atp6v1c1	10090.ENSMUSP00000029900	10090.ENSMUSP0000002290	0	0	0	0	0.51	0.632	0.9	0.76	0.995
Atp6v0d2	Atp6v1a	10090.ENSMUSP00000029900	10090.ENSMUSP0000011031	0.172	0	0	0	0.349	0.609	0.9	0.822	0.995
Atp6v1a	Atp6v1c2	10090.ENSMUSP00000110314	10090.ENSMUSP0000002088	0	0	0	0	0.435	0.727	0.9	0.793	0.996
Atp6v1a	Atp6v1c1	10090.ENSMUSP00000110314	10090.ENSMUSP0000002290	0	0	0	0	0.65	0.796	0.9	0.846	0.998
Atp6v1c1	Atp6v1c2	10090.ENSMUSP00000022904	10090.ENSMUSP0000002088	0	0	0	0.961	0	0.324	0.8	0.681	0.862
Hvcn1	Kcnab2	10090.ENSMUSP00000072401	10090.ENSMUSP0000012505	0	0	0	0	0.054	0	0.9	0.075	0.904

Supplementary Table 4. List of primers used

Gene symbol	Gene name	Catalogue number (Thermo Fisher)
Ctsk	Cathepsin K	Mm00484039_m1
Oscar	Oscar	Mm00558665_m1
Elmo1	Elmo1	Mm00519109_m1
Itgb3	Integrin b3	Mm00443980_m1
Itgb5	Integrin b5	Mm00439825_m1
Hprt1	Hprt1	Mm00446968_m1
