# Supplementary material for: ELMO1 signaling is a promoter of osteoclast function and bone loss
Source: Nat Commun. 2021 Aug 17;12:4974. doi: 10.1038/s41467-021-25239-6 (PMC8371122; doi:10.1038/s41467-021-25239-6)
Supplement: Supplementary file 2 — Description of Additional Supplementary Files [file 41467_2021_25239_MOESM2_ESM.docx]

Description of Additional Supplementary Files

Title: Supplementary Movie 1.

Description: Reduced bone erosions in *Elmo1^–/–^*DBA/1J mice during chronic CIA.
