# Supplementary material for: ELMO1 signaling is a promoter of osteoclast function and bone loss
Source: Nat Commun. 2021 Aug 17;12:4974. doi: 10.1038/s41467-021-25239-6 (PMC8371122; doi:10.1038/s41467-021-25239-6)
Supplement: Supplementary file 3 — Supplementary Movie 1 [file 41467_2021_25239_MOESM3_ESM.pptx]

## Slide 1
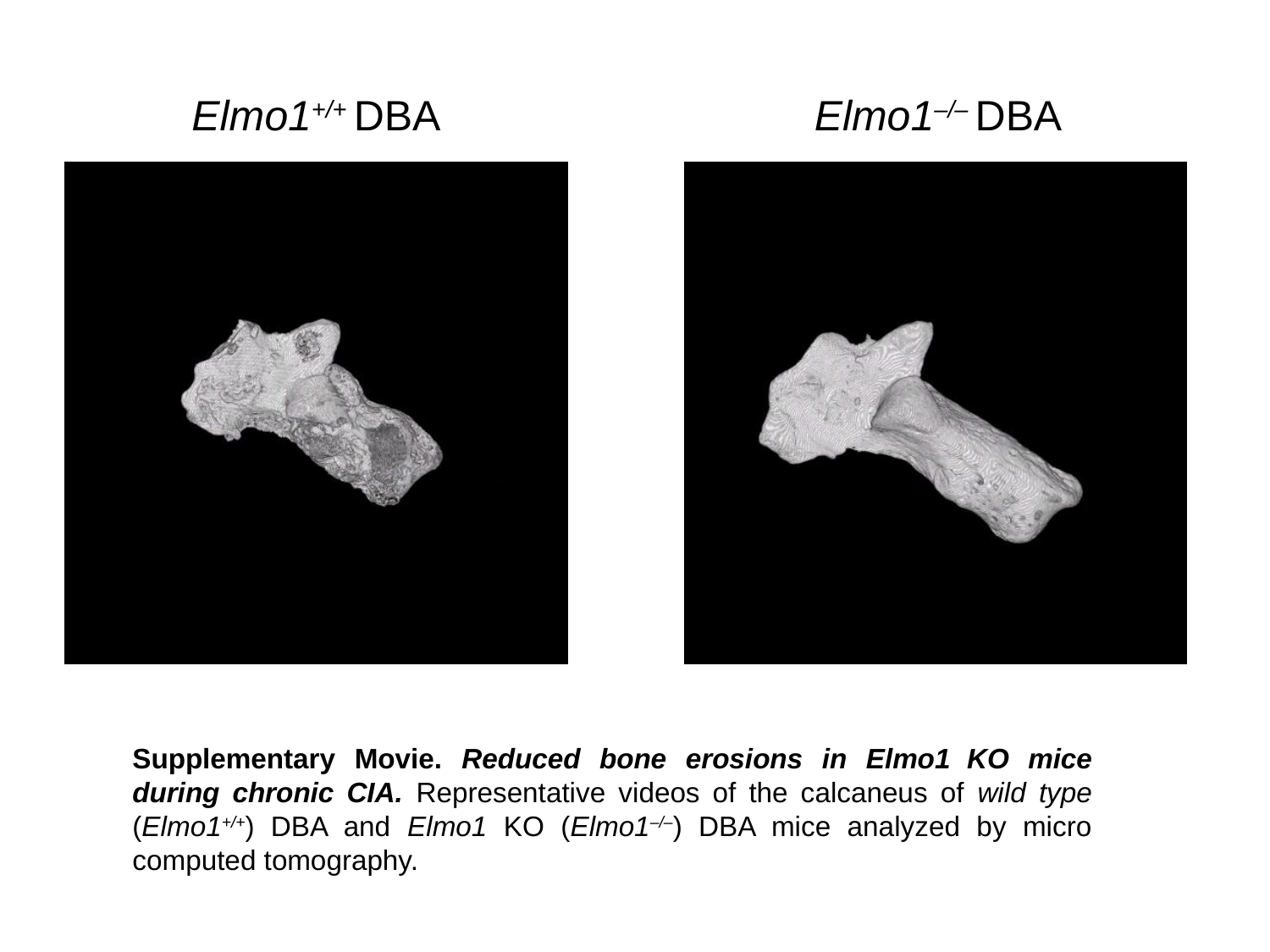

Elmo1+/+ DBA
Elmo1–/– DBA
Supplementary Movie. Reduced bone erosions in Elmo1 KO mice during chronic CIA. Representative videos of the calcaneus of wild type (Elmo1+/+) DBA and Elmo1 KO (Elmo1–/–) DBA mice analyzed by micro computed tomography.
