# Supplementary material for: ELMO1 signaling is a promoter of osteoclast function and bone loss
Source: Nat Commun. 2021 Aug 17;12:4974. doi: 10.1038/s41467-021-25239-6 (PMC8371122; doi:10.1038/s41467-021-25239-6)
Supplement: Supplementary file 4 — Reporting Summary [file 41467_2021_25239_MOESM4_ESM.pdf]

nature research

Corresponding author(s): Kodi S. Ravichandran, Sanja Arandjelovic

Last updated by author(s): 07-07-21

Reporting Summary

Nature Research wishes to improve the reproducibility of the work that we publish. This form provides structure for consistency and transparency in reporting. For further information on Nature Research policies, see our Editorial Policies and the Editorial Policy Checklist.

Statistics

For all statistical analyses, confirm that the following items are present in the figure legend, table legend, main text, or Methods section.

n/a	Confirmed	
☐	☒	The exact sample size (*n*) for each experimental group/condition, given as a discrete number and unit of measurement
☐	☒	A statement on whether measurements were taken from distinct samples or whether the same sample was measured repeatedly
☐	☒	The statistical test(s) used AND whether they are one- or two-sided<br>*Only common tests should be described solely by name; describe more complex techniques in the Methods section.*
☒	☐	A description of all covariates tested
☒	☐	A description of any assumptions or corrections, such as tests of normality and adjustment for multiple comparisons
☐	☒	A full description of the statistical parameters including central tendency (e.g. means) or other basic estimates (e.g. regression coefficient) AND variation (e.g. standard deviation) or associated estimates of uncertainty (e.g. confidence intervals)
☐	☒	For null hypothesis testing, the test statistic (e.g. *F*, *t*, *r*) with confidence intervals, effect sizes, degrees of freedom and *P* value noted<br>*Give P values as exact values whenever suitable.*
☒	☐	For Bayesian analysis, information on the choice of priors and Markov chain Monte Carlo settings
☒	☐	For hierarchical and complex designs, identification of the appropriate level for tests and full reporting of outcomes
☒	☐	Estimates of effect sizes (e.g. Cohen's *d*, Pearson's *r*), indicating how they were calculated

Our web collection on statistics for biologists contains articles on many of the points above.

Software and code

Policy information about availability of computer code

Data collection	FACS Diva v8.0, Attune Nxt Software v2.7, Volocity v6.3, Proteome Discoverer v.2.2.0.388, Mascot v.2.6.0, StepOnePlus v2.3, NextSeq System Suite v1.4 for the Illumina NextSeq v500, Lunar Piximus v2.10, HECTOR, EVOS FL Auto.
Data analysis	GraphPad Prism v5, v6 and v7, ImageLab v6.0, R v3.3.2, FlowJo v10, Volocity v6.3, ImageJ v1.440, Proteome Discoverer v2.1.1.21, Lunar Piximus v2.10, Scanco IPL v5.15, Octopus Reconstruction, VGStudioMAX, EVOS FL Auto, SPSS v.22, Cell Profiler v3.1.9.

For manuscripts utilizing custom algorithms or software that are central to the research but not yet described in published literature, software must be made available to editors and reviewers. We strongly encourage code deposition in a community repository (e.g. GitHub). See the Nature Research guidelines for submitting code & software for further information.

Data

Policy information about availability of data

All manuscripts must include a data availability statement. This statement should provide the following information, where applicable:

- Accession codes, unique identifiers, or web links for publicly available datasets
- A list of figures that have associated raw data
- A description of any restrictions on data availability

The sequencing data generated in this study have been deposited in the GEO database under accession code GSE164826 (http://www.ncbi.nlm.nih.gov/geo/query/acc.cgi?acc=GSE164826). The proteomics data generated in this study have been deposited in the ProteomeXchange under accession code PXD023578 (http://proteomecentral.proteomexchange.org/cgi/GetDataset?ID=PXD023578). Source data are provided with this paper.

April 2020

Field-specific reporting

Please select the one below that is the best fit for your research. If you are not sure, read the appropriate sections before making your selection.

- [x] Life sciences
- [ ] Behavioural & social sciences
- [ ] Ecological, evolutionary & environmental sciences

For a reference copy of the document with all sections, see nature.com/documents/nr-reporting-summary-flat.pdf

Life sciences study design

All studies must disclose on these points even when the disclosure is negative.

Sample size	No statistical tests were used to predetermine sample size. For in vivo experiments, sample sizes were determined based on the numbers required to achieve statistical significance using non-parametric statistics. For in vitro experiments, littermates were used and experiments repeated to ensure reproducibility.
Data exclusions	Statistical tests for outliers are routinely performed. No data were excluded for our analyses.
Replication	Consistent results obtained from at least two or three biological replicates per experiment were used in the manuscript. All attempts at replication were successful.
Randomization	Allocation of mice was random in all in vivo experiments, taken from littermates. Littermates were also used for in vitro analyses.
Blinding	The investigators were blinded to allocation during all in vivo experiments and outcome assessments. In vitro analysis of osteoclast resorption pits number/size was also performed by an investigator blinded to the culture genotypes. Blinding was not relevant for immunoblot and immunofluorescence analyses.

Reporting for specific materials, systems and methods

We require information from authors about some types of materials, experimental systems and methods used in many studies. Here, indicate whether each material, system or method listed is relevant to your study. If you are not sure if a list item applies to your research, read the appropriate section before selecting a response.

Materials & experimental systems

n/a	Involved in the study	
[ ]	[x]	Antibodies
[ ]	[x]	Eukaryotic cell lines
[x]	[ ]	Palaeontology and archaeology
[ ]	[x]	Animals and other organisms
[x]	[ ]	Human research participants
[x]	[ ]	Clinical data
[x]	[ ]	Dual use research of concern

Methods

n/a	Involved in the study	
[x]	[ ]	ChIP-seq
[ ]	[x]	Flow cytometry
[x]	[ ]	MRI-based neuroimaging

Antibodies

Antibodies used	Antibodies used for flow cytometry were anti-CD16/CD32 (eBioscience clone 93), anti-CD11b (eBioscience clone M1/70) and anti-F4/80 (eBioscience clone BM8). All flow cytometry antibodies were used following the manufacturer's instructions. Antibodies used for immunoblotting were anti-ELMO1 rabbit polyclonal antibody (made in-house, 1:1,000 dilution), anti-ERK2 (Santa Cruz, goat polyclonal, 1:3,000 dilution), anti-beta-ACTIN-HRP (Sigma clone AC-15, 1:10,000 dilution), and anti-Rac1 (Cytoskeleton #ARC03). Antibody used for immunoprecipitation was anti-ELMO1 mouse monoclonal antibody (made in-house, used at 5 micrograms per milligram of total protein extract). All antibody clones, manufacturers and dilutions used are listed in the Methods section of the manuscript.
Validation	All commercial antibody lots are routinely tested by the manufacturers, and each product comes with a certificate of analysis from the indicated vendor stating that the product has met all quality control standards. Any new antibody used for flow cytometry or western blotting was tested with an appropriate isotype control or protein-deficient cells.

Eukaryotic cell lines

Policy information about cell lines

Cell line source(s)	L929 cell lines were acquired from the ATCC and are available upon request.

Authentication	Morphological shape of cell lines and expression of specific cell surface markers used was regularly monitored via microscopic examination and flow cytometry.
Mycoplasma contamination	All cell lines used in the laboratory are regularly tested for mycoplasma contamination. Additionally, all media and serum lots used are regularly tested as well. All materials used in this study tested negative for mycoplasma contamination.
Commonly misidentified lines (See ICLAC register)	No commonly misidentified cell lines were used.

Animals and other organisms

Policy information about studies involving animals; ARRIVE guidelines recommended for reporting animal research

Laboratory animals	All animals and animal-derived materials are reported in the Methods section. Animals were maintained according to IACUC and UVa animal facility standards, including ad libitum feeding. C57BL/6J, DBA/1J, NOD, Cx3cr1-Cre, Opg–/– and RosaYFP mice were obtained from Jackson Laboratories. KRN TCR transgenic mice were a gift from Dr. Diane Mathis at the Harvard Medical School. Elmo1fl/fl and Elmo1–/– mice have been generated in our laboratory and were described previously (see Methods). Age- and sex-matched littermate control animals were used for all experiments, and both males and females were assessed. Age of animals used for specific experiments is described either directly in the manuscript or in the Methods section.
Wild animals	This study did not involve any wild animals.
Field-collected samples	This study did not involve field-collected samples.
Ethics oversight	All animal procedures were approved by and performed according to guidelines of the Institutional Animal Care and Use Committee (IACUC) at the University of Virginia.

Note that full information on the approval of the study protocol must also be provided in the manuscript.

Flow Cytometry

Plots

Confirm that:

- [x] The axis labels state the marker and fluorochrome used (e.g. CD4-FITC).
- [x] The axis scales are clearly visible. Include numbers along axes only for bottom left plot of group (a 'group' is an analysis of identical markers).
- [x] All plots are contour plots with outliers or pseudocolor plots.
- [x] A numerical value for number of cells or percentage (with statistics) is provided.

Methodology

Sample preparation	All sample preparation is reported in the Methods section. Peritoneal exudates were obtained via cold PBS flush. All samples were filtered prior to staining. Samples were kept on ice during staining and collection. All fluorescent antibodies were aliquoted in a sterile hood with minimal light exposure. Staining of samples were protected from light throughout.
Instrument	Data were collected on a FACS Canto I (Becton Dickinson) or the Attune Nxt (Thermo Fisher) flow cytometers.
Software	Data were collected analyzed with FlowJo v10 (Treestar, Inc).
Cell population abundance	Purity of isolated samples was obtained by antibody stain and FACS. Sample purity was greater than 95% in all experiments.
Gating strategy	Standard lymphocyte gates were applied, following by doublet exclusion using FSC-AxW and SSC-AxW. In our studies, macrophages were gated using a combination CD11b and F4/80.

- [x] Tick this box to confirm that a figure exemplifying the gating strategy is provided in the Supplementary Information.
